# Characterization, Stability, and In Vivo Efficacy Studies of Recombinant Human CNTF and Its Permeation into the Neural Retina in Ex Vivo Organotypic Retinal Explant Culture Models

**DOI:** 10.3390/pharmaceutics12070611

**Published:** 2020-06-30

**Authors:** Jaakko Itkonen, Ada Annala, Shirin Tavakoli, Blanca Arango-Gonzalez, Marius Ueffing, Elisa Toropainen, Marika Ruponen, Marco G. Casteleijn, Arto Urtti

**Affiliations:** 1 Drug Research Program, Faculty of Pharmacy, University of Helsinki, Viikinkaari 5 E, 00790 Helsinki, Finland; shirin.tavakoli@helsinki.fi (S.T.); marco.casteleijn@vtt.fi (M.G.C.); 2 School of Pharmacy, University of Eastern Finland, Yliopistonranta 1, 70211 Kuopio, Finland; a.k.a.annala@uu.nl (A.A.); elisa.toropainen@uef.fi (E.T.); marika.ruponen@uef.fi (M.R.); 3 Utrecht Institute for Pharmaceutical Science, Utrecht University, David de Wiedgebouw, Universiteitsweg 99, 3584 CG Utrecht, The Netherlands; 4 Institute for Ophthalmic Research, Centre for Ophthalmology, University of Tübingen, Elfriede-Aulhorn-Strasse 7, D-72076 Tübingen, Germany; blanca.arango-gonzalez@klinikum.uni-tuebingen.de (B.A.-G.); marius.ueffing@uni-tuebingen.de (M.U.); 5 VTT Technical Research Centre of Finland Ltd., Solutions for Natural Resources and Environment, Tietotie 2, Espoo, P.O. Box 1000, FI-02044 VTT, Finland; 6 Laboratory of Biohybrid Technologies, Institute of Chemistry, St. Petersburg State University, Universitetskii pr. 26, Peterhoff, 198504 St. Petersburg, Russia

**Keywords:** retinal penetration, neuroprotection, protein aggregation, stability, intravitreal delivery, CNTF

## Abstract

Ciliary neurotrophic factor (CNTF) is one of the most studied neuroprotective agents with acknowledged potential in treating diseases of the posterior eye segment. Although its efficacy and mechanisms of action in the retina have been studied extensively, it is still not comprehensively understood which retinal cells mediate the therapeutic effects of CNTF. As with therapeutic proteins in general, it is poorly elucidated whether exogenous CNTF administered into the vitreous can enter and distribute into the retina and hence reach potentially responsive target cells. Here, we have characterized our purified recombinant human CNTF (rhCNTF), studied the protein’s in vitro bioactivity in a cell-based assay, and evaluated the thermodynamic and oligomeric status of the protein during storage. Biological activity of rhCNTF was further evaluated in vivo in an animal model of retinal degeneration. The retinal penetration and distribution of rhCNTF after 24 h was studied utilizing two ex vivo retina models. Based on our characterization findings, our rhCNTF is correctly folded and biologically active. Moreover, based on initial screening and subsequent follow-up, we identified two buffers in which rhCNTF retains its stability during storage. Whereas rhCNTF did not show photoreceptor preservative effect or improve the function of photoreceptors in vivo, this could possibly be due to the used disease model or the short duration of action with a single intravitreal injection of rhCNTF. On the other hand, the lack of in vivo efficacy was shown to not be due to distribution limitations; permeation into the retina was observed in both retinal explant models as in 24 h rhCNTF penetrated the inner limiting membrane, and being mostly observed in the ganglion cell layer, distributed to different layers of the neural retina. As rhCNTF can reach deeper retinal layers, in general, having direct effects on resident CNTF-responsive target cells is plausible.

## 1. Introduction

Progressive diseases of the posterior eye segment, and in particular those affecting the retina, are among the most common causes of visual impairment and blindness [1]. Cataract and glaucoma, for instance, are the two leading causes of blindness globally, whereas age-related macular degeneration (AMD) is the most common culprit for legal blindness in the aged population in high-income countries, affecting approximately 10% of the population over 60 years of age [2,3]. Degenerative diseases of the posterior eye segment, including conditions such as diabetic retinopathy and retinitis pigmentosa (RP), are often age-related, and their prevalence is growing in the aging populations.

Pharmaceuticals that target retinal disease pathogenesis are currently available only for the treatment of diabetic macular edema and the wet form of AMD, conditions that feature pathological angiogenesis in the retina, which is driven by the overexpression of pro-angiogenic factors [3]. Whereas other factors are involved, the vascular endothelial growth factors (VEGFs) are recognized as the main mediators responsible for the pathological neovascularization in the posterior eye segment [2]. Interfering with VEGFs’ binding to their receptors (VEGFRs) inhibits the VEGF-triggered actions of these receptor tyrosine kinases. As such, VEGF-blocking as a treatment strategy is most commonly achieved with therapeutic proteins that bind and neutralize VEGF [1].

Although neovascular diseases result in retinal neurodegeneration in particular during advanced stages, anti-VEGF treatments rescue retinal neurons indirectly, and their ocular use is limited to treating neovascular diseases only. Effective treatments affecting retinal degeneration directly are still lacking [4]. As advanced posterior segment diseases in general feature degeneration and loss of retinal neurons, with photoreceptor loss ultimately accounting for the experienced loss of vision, targeting the involved pathways by means of direct neuroprotection is considered a more universal approach to combat retinal neurodegeneration [4,5]. Several growth factors and neurotrophic factors, e.g., brain-derived neurotrophic factor, fibroblast growth factors, glial cell-line derived factor, and nerve growth factor have been studied in preclinical animal models for their neuroprotective potential in oculo [6,7]. In addition to these, the ciliary neurotrophic factor (CNTF) is arguably the most studied and has progressed the furthest in clinical trials [4,8].

CNTF has an approximate molecular mass of 23 kDa and a four α-helix bundle tertiary structure [9,10]. It belongs to the interleukin-6 (IL-6) family of neuropoietic cytokines and exerts neurotrophic effects on a variety of neurons. Lacking a signal sequence for secretion, the mechanism by which CNTF gets released from cells is still unknown and postulated to occur upon cellular injury [8,11]. CNTF elicits its actions through a receptor complex consisting of the ligand-specific α-receptor CNTFRα, and β-receptors glycoprotein 130 (gp130) and leukemia inhibitory factor receptor β (LIFRβ) [9,11]. Lacking transmembrane and cytoplasmic domains, CNTFRα is anchored to the cell membrane by a glycosylphosphatidylinositol linker; cleavage of this linker can release soluble receptor, sCNTFRα, rendering cells expressing just gp130 and LIFRβ also capable of responding to CNTF [9].

In the retina, CNTF expression spans all layers and occurs in several cell types, such as retinal pigment epithelium (RPE), and particularly in glial cells such as astrocytes and Müller cells [8,12,13]. CNTF protein expression is upregulated in response to, e.g., stress and injury [12,14,15]. CNTFRα expressing retinal cells include astrocytes, Müller cells, retinal ganglion cells (RGC), rod and cone photoreceptors, and RPE [8,13,15,16,17,18,19]. Whereas both murine and rat central nervous system (CNS) microglia express CNTFRα [20,21,22], it is not fully clear whether local resident microglia do so in the retina, although they have nonetheless been shown to respond to CNTF [23].

Whereas CNTFRα expressing retinal cells are potentially responsive to CNTF, it is not established whether all of CNTF’s effects on different cell types are, in fact, direct or mediated indirectly by specific cells [8]. Observations on species differences in retinal CNTFRα expression in part complicate interpreting and extrapolating findings from one species to another [16]. Additionally, non-CNTFRα expressing retinal cells expressing the common signal transduction components could nonetheless be conferred responsive to CNTF by sCNTFRα [8,24]. Currently, CNTF’s neuroprotective effects on RGCs and photoreceptors are thought to take place (mostly) indirectly via Müller cells [8,12,25], which are postulated to respond to CNTF stimulation by expressing and releasing other cytokines and neurotrophic factors that relay CNTF’s neuroprotective effects to other retinal cells [15,18,25,26,27,28].

Although the mechanisms and mediators of the biological effects of CNTF are still not fully resolved, its supportive and neuroprotective actions against retinal damage and degeneration have been demonstrated in various animal models of retinal disease [5,8]. The photoreceptor preservative effect related to upregulation of endogenous CNTF after mechanical or light-induced damage is well-reported in literature [14,29,30], and multiple teams working on viral-mediated gene therapies or sustained release delivery systems have demonstrated the protective effects of CNTF in different animal models of retinal degeneration [31,32,33,34,35,36]. While intravitreally administered CNTF has been shown to protect photoreceptors from light-induced damage, the preservative effect on photoreceptor degeneration caused by a genetic defect has not been as clear and seems, to some extent, to depend on the species and disease model [36,37,38,39]. In S334ter-3 rats, intravitreal (IVT) injection of CNTF improved the retinal morphology, and especially the cone photoreceptors seemed to benefit from the treatment [35,40]. In a Royal College of Surgeons (RCS) rat, single subretinal injection of recombinant CNTF showed a long-term photoreceptor preservative effect up to 36 days [33]. However, the efficacy of intravitreally administered CNTF has not been previously studied in this animal model.

Certain aspects of CNTF have been inadequately described in the literature. Whereas hCNTF variants with improved stability were in development for CNS delivery [41], publications reporting on the formulation and related stabilization efforts on CNTF are scarce. Previously, we reported on the optimized soluble expression and purification of our recombinant human CNTF (rhCNTF) [42]. Here, we describe further characterization and stability studies of rhCNTF. Although the Müller glia cells are considered the primary mediators of CNTF’s actions in the retina, direct effects on other cells cannot be ruled out as CNTF-responsive as well as CNTFRα-expressing cells have been identified in several retinal layers [8]. However, little is known about the retinal penetration and distribution of exogenously administered CNTF, although the inner limiting membrane (ILM) at the vitreoretinal interface has been postulated to be the biggest limitation to CNTF’s entry into the retina [27]. Here, we evaluate the efficacy of intravitreally administered rhCNTF in vivo in RCS rat and report on the retinal penetration and distribution of our rhCNTF in organotypic ex vivo retinal explant cultures.

## 2. Materials and Methods

### 2.1. Protein Production

The features and construction of the expression plasmid pOPINF-hCNTF as well as the expression and purification of soluble recombinant His_6_-hCNTF, from now on referred to as rhCNTF, have been described earlier [42,43]. Aside from expression in Rosetta^TM^ 2(DE3)pLysS (Novagen, Merck KGaA, Darmstadt, Germany) *E. coli* cells using the Overnight Express™ Instant TB auto-induction culture medium (Novagen, Merck) [44], rhCNTF was also expressed in the aforementioned cells using EnPresso^®^ B growth system [45] (BioSilta Oy, Oulu, Finland) according to the manufacturer’s specifications.

Protein purification was carried out as describer earlier [42] and is described in more detail in the Supplement. For further studies, the purified protein was kept on ice at 4 °C as well as snapfrozen with liquid N_2_ for storage at −80 °C.

### 2.2. rhCNTF In Vitro Bioactivity Study

The correct function, and thus indirectly the proper folding of purified rhCNTF, was demonstrated earlier in an enzyme-linked immunosorbent assay (ELISA) binding study with the cognate receptor CNTFRα [42]. To ensure that rhCNTF can trigger downstream signaling and hence biological responses, an in vitro cell study was performed.

#### 2.2.1. Cell Culture

TF-1-CN5a.1 (product CRL-2512™, American Type Culture Collection, ATCC^®^, Manassas, VA, USA) cells were obtained from LGC Standards (Teddington, UK). The cells were maintained in complete growth medium of Roswell Park Memorial Institute (RPMI) 1640 medium, 2 mM L-glutamine, 1.5 g/L sodium bicarbonate, 4.5 g/L glucose, 10 mM HEPES, and 1.0 mM sodium pyruvate (RPMI-1640, ATCC modification; Gibco^TM^, Thermo Fisher Scientific, Waltham, MA, USA) supplemented with 10% fetal bovine serum (Gibco^TM^, Thermo Fisher Scientific), 2 ng/mL human granulocyte macrophage colony-stimulating factor (hGM-CSF) (Sigma-Aldrich, St. Louis, MO, USA), 0.4 mg/mL G-418 (Calbiochem^TM^, Merck), and 100 U/mL penicillin – 100 µg/mL streptomycin (Gibco^TM^, Thermo Fisher Scientific). The cells were maintained as stationary suspension cultures in non-treated Nunc™ EasyFlask™ 75 cm^2^ flasks (Thermo Fisher Scientific) in a fully humidified 5% CO_2_ atmosphere at 37 °C. Cell number and viability were determined using trypan blue.

#### 2.2.2. Cell Proliferation Assay

The bioactivity of the purified rhCNTF was verified by measuring cell proliferation in a 5-bromo-2’-deoxyuridine (BrdU) incorporation assay [46], using the Cell Proliferation ELISA BrdU kit (Roche Diagnostics, Mannheim, Germany) according to manufacturer’s specifications. First, 50 µL of serial dilutions of purified rhCNTF in assay growth medium, i.e., complete growth medium without hGM-CSF and G-418, were prepared and added in triplicates to the wells of CELLSTAR^®^ 96-well microplates for suspension cells (Greiner Bio-One GmbH, Kremsmünster, Austria). Cultured TF-1.CN5a.1 cells were first centrifuged and washed with RPMI-1640 to remove hGM-CSF, followed by resuspension in assay growth medium. Cells were then seeded at 1.0 × 10^4^ cells in 50 µL/well (final rhCNTF concentrations 24 fg/mL–100 ng/mL). Cells cultured in assay growth medium with and without 2 ng/mL hGM-CSF were used as positive and negative controls, respectively. Treated cells were incubated at 37 °C, 5% CO_2_ for 48 h.

After incubation, 10 µL of 100 µM BrdU labeling solution was added to the wells and the cells (were) incubated for an additional 2 h, allowing for the incorporation of BrdU into the synthesized DNA during cell proliferation. After labeling, the suspended cells were pelleted by centrifugation at 300× *g* for 10 min, the media removed from the wells gently by pipetting, and the cells dried at 60 °C for 60 min. Then, 200 µL of FixDenat was added to each well for cell fixation and DNA denaturation and removed by pipetting after 30 min incubation at room temperature. Incubation with a peroxidase-conjugated anti-BrdU antibody was carried out for 90 min at room temperature to allow for binding to the incorporated and now exposed BrdU. Antibody solutions were then aspirated, and the cells were washed 4 times with 200 µL/well washing solution. After incubation with the 3,3′,5,5′-tetramethylbenzidine substrate solution 15 min, the absorbances were determined at 370 nm and 492 nm on a microplate reader (Varioskan^®^ Flash; Thermo Fisher Scientific).

### 2.3. rhCNTF Characterization and Stability Studies

Upon visual inspection, we had observed precipitation—likely due to protein aggregation—taking place upon freeze-thawing with previously purified rhCNTF stored in 100 mM NaH_2_PO_4_, 50 mM NaCl, pH 8.0, 1 mM dithiothreitol (DTT) buffer at −80 °C resulting in a loss of approximately a third of stored protein (not shown). There was, hence, a clear need to find a more suitable storage buffer to stabilize the protein.

To gain insight on the structure and stability of rhCNTF, the protein was subjected to experiments using circular dichroism, differential scanning fluorimetry, and dynamic light scattering to characterize, e.g., secondary structure, thermal unfolding, and oligomeric state.

#### 2.3.1. Circular Dichroism

Circular Dichroism (CD) spectroscopy was utilized to study the secondary structure and folding of the purified rhCNTF. Far-UV CD spectra (190–260 nm) of rhCNTF (0.2 mg/mL) samples desalted with 5 mM phosphate buffer were obtained in a 0.1 cm quartz cuvette at 25 °C with a Chirascan^TM^-Plus CD Spectrometer (Applied Photophysics Ltd., Leatherhead, Surrey, UK). Background spectrum of the buffer was recorded and subtracted from subsequent triplicate spectral scans with the rhCNTF samples, with the average spectrum plotted. Pro-Data Viewer (Applied Photophysics) was used for data collection and handling. Data were normalized to protein concentrations and expressed in units of mean residue molar ellipticity. The BeStSel algorithm was also used for secondary structure determination from CD spectra [47].

Thermal denaturation and unfolding of rhCNTF was studied during thermal ramping from 25 to 92 °C (1 °C/min) with far-UV CD (190–260 nm) measurements every 2 °C. Values for the onset (T_onset_) and midpoint (T_m_) of unfolding were determined by plotting measured ellipticity at 222 nm, a signal proportional to α-helical content, as a function of temperature.

#### 2.3.2. ThermoFluor

Differential scanning fluorimetry, commonly known as ThermoFluor, is a thermal shift assay based on an increase in the fluorescence of several non-specific protein-binding dyes upon their binding to certain regions and residues that become accessible during protein unfolding [48,49]. From the recorded fluorescence signal, parameters such as the T_h_—the temperature of hydrophobic exposure—and other information of the thermal unfolding of proteins can be extracted. Here, based on observations that higher T_h_ correlates with higher conformational stability in formulation, different conditions were tested for their effects on the T_h_ of rhCNTF to screen for suitable buffers to stabilize the protein.

Buffers were pipetted to semi-skirted qPCR 96-well plates (Agilent Technologies, Inc., Santa Clara, CA, USA) while rhCNTF protein samples and SYPRO^®^ Orange (Thermo Fisher Scientific) were applied as droplets on opposing edges of the wells to prevent premature interactions. Wells were sealed with Bio-Seal 7 Transparent Adhesive Seals for PCR plates (BIOplastics, Landgraaf, The Netherlands), the plates were quickly centrifuged to bring all components together in a total reaction volume of 25 µL, mixed by gentle vortexing, and again centrifuged. The measurements were carried out in a Stratagene Mx3005P qPCR instrument (Agilent Technologies). Samples were heated from 25 to 95 °C at a rate of 1 °C per 30 s. Fluorescence readouts were taken at every °C step using 492 nm and 610 nm as the excitation and emission wavelengths, respectively. Measurements were analyzed and T_h_ values determined with MxPro qPCR software (Agilent Technologies).

ThermoFluor was first carried out on a grid of varying rhCNTF and dye concentrations as a pre-screening to optimize protein/dye concentrations [50], and the subsequent measurements carried out with the best match. To study the effects of buffer, ionic strength, and pH on rhCNTF thermostability, 94 different conditions were screened for increased rhCNTF T_h_ (Table S1). Arising trends in the T_h_ as a surrogate for conformational stability were then followed with rhCNTF stored in chosen buffers at 4 °C and −80 °C.

#### 2.3.3. Dynamic Light Scattering

The formation and presence of protein aggregates was assessed using dynamic light scattering (DLS). DLS measurements were carried out using Zetasizer APS (Malvern Panalytical Ltd., Malvern, Worcestershire, UK) with a 830 nm laser source using the Zetasizer Software (Malvern Panalytical) for data acquisition and analysis. Purified rhCNTF samples in the chosen buffers were diluted to 1.0 mg/mL and filtered using 0.22 µm polyethersulfone (PES) membrane filters to remove large particulates. Each sample was measured in triplicate at 2 °C. The size and hydrodynamic radius (R_h_) distributions of detected particles were obtained by measuring and integrating the intensity of the scattered light, whereas for follow-up and comparing measurements, parameters were derived both via intensity and volume analysis.

Samples kept on ice at 4 °C after purification as well as snap-frozen samples stored at −80 °C were measured at different time points to assess for changes and trends in the R_h_. For the cryo-stored samples, measurements with thawed unmixed sample as well as with the supernatant from centrifuged (21,100× *g*, 30 min at 4 °C) thawed rhCNTF were carried out for comparison.

The thermal aggregation behavior of rhCNTF was also studied with DLS by carrying out R_h_ measurements with freshly purified rhCNTF in both buffers during thermal ramping. To determine the onset temperature (T_agg_), i.e., the temperature at which proteins start to associate and aggregate and at which scattering from HMW species intensifies, samples were heated from 2 to 60 °C and particle size measurements taken at each °C step. The measured Z-average (in nm) was plotted as a function of temperature.

### 2.4. rhCNTF In Vivo Efficacy Study

#### 2.4.1. Animals

The photoreceptor preservative effect of rhCNTF was assessed in vivo after an IVT bolus injection using Royal College of Surgeons (RCS) rat as an animal model of retinal degeneration. The animals were 18 or 21 days old, both male and female. Animals were kept in controlled conditions (12 h light/dark cycle, temperature 21 ± 2 °C, humidity 55 ± 15%, ventilation 15 times per hour), housed with a maximum of 5 animals per stainless-steel cage (28.5 cm × 48.5 cm × 20 cm) with food and water provided ad libitum. The cages were changed twice a week and provided with sufficient bedding, nesting material, and enrichment. All procedures were performed in accordance to the European and national legislation on the protection of animals used for scientific or educational purposes (Directive 2010/63/EU, Act 497/2013, Decree 564/2013; ESAVI/6791/04.10.07/2013).

#### 2.4.2. Intravitreal Injections

Stock solution of rhCNTF protein in buffer M (100 mM MES, 500 mM NaCl, pH 7.0) was thawed on ice prior to purification from aggregates by centrifuging (13,000× *g*, 15 min, +2 °C, Heraeus Biofuge Fresco, Heraeus Instruments). The supernatant was diluted to 500 ng/mL with buffer M with 1 mM DTT, sterile filtered through 0.2 µm PTFE membrane (Acrodisc^®^ Syringe Filter CR13; PALL Corporation, Port Washington, NY, USA), stored in 4 °C, and used within one week. The final concentrations used for experiments were diluted with buffer M prior to injection from the stock solution.

Animals were weighed and anaesthetized with intraperitoneal (i.p.) injection of 1 mg/kg of medetomidine (Domitor vet 1 mg/mL, Orion Corporation, Espoo, Finland) and 75 mg/kg of ketamine (Ketaminol vet 50 mg/mL, Intervet International B.V), diluted with 0.9% sodium chloride solution (Natriumklorid Braun, B.Braun, Germany). The pupils were dilated with tropicamide (Oftan Tropicamid 5 mg/mL, Santen Pharmaceutical Co., Ltd., Osaka, Japan), which was allowed to affect for 10 min to achieve full dilation of the pupils. Finally, carbomer eye gel (Viscotears, Novartis International GA, Basel, Switzerland) was added to the eyes to prevent corneal desiccation.

In the 1st study set, 21-day-old animals received single IVT injection (2 µL) of 1 µg rhCNTF (n = 4), 500 ng rhCNTF (n = 5) and 250 ng rhCNTF (n = 4) or buffer M (n = 4) into one eye, while the contralateral eye was left untreated and served as an internal control.

In the 2nd study set, 18-day-old animals received single IVT injection (2 µL) of 1 µg rhCNTF (n = 7), or buffer M (n = 6) to one eye, while the contralateral eye was left untreated and served as an internal control. Untreated naïve control animals, NControl (n = 7) served as external control.

Injection was performed with 5 µL Hamilton^®^ syringe (Hamilton Company, Inc., Reno, NV, USA) using a sharp point 34-gauge needle. Incision was done approximately 1 mm from the limbus, at a 45-degree angle, towards the back of the vitreous. Eyes of the animals were treated with antibiotic ointment after injection (Dexamethasone 1 mg/g, chloramphenicol 2 mg/g, Oftan Dexa-Chlora, Santen Pharmaceutical Co.) to prevent inflammation and to minimize solution efflux from the vitreous. Anesthesia was reversed with i.p. injection of atipamezole (Antisedan vet 5 mg/mL, Orion Corporation.). The animals were monitored during anesthesia and recovery from procedure was ensured. Any animals with welfare issues, visible injuries to the injected eye, or other signs of unsuccessful operation were determined as outliers and eliminated from the experiment.

#### 2.4.3. Electroretinogram (ERG) Recording

Full-field ERG was recorded 1- and 2-weeks post-treatment. Animals were weighed and dark-adapted overnight, for a minimum of 12 h. All procedures were carried out under dim red light. The animals were anesthetized, pupils dilated, and carbomer gel added to the eyes as previously described. The animals were placed on a heated surface during the measurements. Rod responses were stimulated in dark-adapted conditions with series of dim blue flashes with increasing intensities (0.003, 0.007, 0.03, and 0.5 cd × s/m^2^, number of sweeps 3, inter-sweep delay 5000 ms), whereas cone responses were stimulated under light-adapted conditions with series of bright white flashes (0.1, 1, 3, 5, 10, and 20 cd × s/m^2^, number of sweeps 12, inter-sweep delay 0 ms) using ColorDome D125 (Diagnosys LLC, Lowell, MA, USA). The retinal responses were detected from the cornea with custom-made circular golden electrodes and recorded using Espion Visual Electrophysiology System V6 (Diagnosys LLC). The resulting retinograms were analyzed using Espion V6 software. The recovery of the animals was monitored for two days after every recording.

#### 2.4.4. Data-Analysis

ERG results were analyzed using non-parametric testing using Microsoft Excel, GraphPad Prism 8, and IBM SPSS software. The parameter distribution between all treatment groups at all flash intensities was analyzed with the Kruskal–Wallis test. The comparison between the treated and untreated contralateral eye, as well as the comparison between the ERG values recorded 1 week and 2 weeks post-injection, was achieved with the Wilcoxon signed-rank test.

#### 2.4.5. Histology

Animals were euthanized at the age of 35 days (2nd study set) or 38 days (1st study set) with CO_2_ overdose followed by perfusion through the heart with 9% sodium chloride. The ocular tissue was pre-fixed in situ by perfusion through the heart by 4% paraformaldehyde (PFA) followed by ocular enucleation and fixation overnight in 4% PFA in 12-well plates in 4 °C. Thereafter, the eyes were rinsed in phosphate-buffered saline (PBS) for 2 to 6 h and dehydrated in the processing machine as described in Table S2 (Shandon Citadel 2000 Tissue Processor, Thermo Fisher Scientific). The eyes were embedded in liquid paraffin (64 °C), cooled to ambient temperature, and 5 µm vertical cross sections were cut close to the papilla (Leica SM2000R, Leica instruments GmbH, Germany or HM 355S Rotary Microtome, Thermo Fisher Scientific). Sections were flattened on warm water bath and collected on microscope slides and treated with hematoxylin and eosin (H&E) staining procedure according to Table S3. Glass slides were covered with DPX plastic and allowed to dry for 18 to 30 h. Sections were imaged using a Zeiss light microscope (Axio Imager M2; Carl Zeiss AG, Oberkochen, Germany) with 20× magnification (EC Plan-NEOFLUAR 20X/0.5 objective, Carl Zeiss AG) using AxioCam MRm (Carl Zeiss AG).

### 2.5. Retinal Penetration of rhCNTF

#### 2.5.1. rhCNTF Fluorescent Labeling

Purified rhCNTF was fluorescently labeled with Alexa Fluor™ 488 Microscale Protein Labeling Kit (Thermo Fisher Scientific) via tetrafluorophenyl ester linkage to free amines according to manufacturer’s instructions. Unreacted dye was separated from labeled protein with provided spin filters and the degree of labeling analyzed with a DropSense16 spectrophotometer (Trinean NV, Gentbrugge, Belgium) according to manufacturer’s instructions, with a calculated degree of labeling (DOL) of 1.16 indicating the average number of dye molecules conjugated to each protein molecule. Labeled rhCNTF was aliquoted and stored on ice until use on rat retinal explant cultures.

Similarly, rhCNTF was fluorescently labeled with NT-647 (NanoTemper Technologies, München, Germany) via *N*-hydroxysuccinimide ester linkage to free amines according to manufacturer’s instructions. Unreacted dye was removed with provided columns and the DOL analyzed with Varian Cary^®^ 50 spectrophotometer (Agilent Technologies) according to manufacturer’s instructions, with a calculated DOL of 0.557 indicating the average number of dye molecules conjugated to each protein molecule. Labeled rhCNTF was aliquoted and stored at −80 °C until use on bovine retinal explant cultures.

#### 2.5.2. Retinal Explant Culture Preparation and rhCNTF Treatment

##### Rat Retinal Explants

Eyes obtained from CD^®^ (SD) IGS rats were used to prepare organotypic retinal explant cultures as described earlier [51,52]. In brief, 5-, 6-, or 8-day-old animals were killed and the eyes enucleated in an aseptic environment. After cleansing with 70% ethanol, the eyes were incubated in basal R16 medium (Invitrogen, Paisley, UK) for 5 min, followed by incubation in pre-warmed 0.12% proteinase K (MP Biomedicals^TM^, Thermo Fisher Scientific) for 15 min at 37 °C. To inactivate proteinase K, the eyes were incubated in basal R16 medium containing 20% fetal bovine serum (Sigma-Aldrich) for 5 min, and finally washed in serum-free basal R16 medium. Dissections were carried out aseptically in a Petri dish containing basal R16 medium. The anterior segment, sclera, choroid, lens, and the vitreous body were carefully removed, leaving only the retina together with the attached RPE. Finally, four relaxing cuts were made, and the retinae flat-mounted with the photoreceptor-side down on culture membrane inserts (0.4 µm; Corning Incorporated, Corning, NY, USA; 3412). Complete R16 medium was placed in the lower compartments of 6-well culture dishes, and the cultures were incubated at 36.5 °C and 5% CO_2_. No antibiotics or antimycotics were used.

Explants were left without treatment for 24 h to allow them to adapt to culture conditions, followed by rhCNTF treatments for the next 24 h; the experiment duration was kept at a minimum to minimize explant deterioration, i.e., to retain the structure of all the retinal layers as close to native as possible. Alexa Fluor™ 488-labeled rhCNTF was prepared in PBS, diluted in basal R16 medium, and sterilized with 0.22 µM PES membrane filter before use. To mimic dosing via IVT and e.g., subretinal injections, treatments were given either apically as 15 µL drops (200 ng dose) carefully applied directly on top of the explant cultures or basolaterally as 4.7 µg/mL in complete medium below the explant in the lower compartment, respectively. Untreated complete R16 medium was used as control.

##### Bovine Retinal Explants

Retinal explant cultures were prepared as described earlier [53]. Fresh bovine eyes obtained from a local slaughterhouse were first cleaned off extra-ocular connective tissues followed by dipping in 20% ethanol. The eye was bisected 10 mm below the limbus, the anterior segment discarded and the vitreous removed, with the remaining posterior eye cup filled with cold CO_2_ independent medium (Gibco™, Thermo Fisher Scientific) and cut into 4 flaps. While submerged in the medium, two circular pieces of the retina were isolated using a biopsy punch and then gently transferred onto moisturized 75 mm Transwell^®^ membrane with the photoreceptor-side down. Explant culture medium (Neurobasal™-A, 2% B-27™ supplement, 2% penicillin–streptomycin, 1% L-glutamine; all Gibco™, Thermo Fisher Scientific) was added below the insert and the explants incubated at 37 °C and 5% CO_2_.

Treatments were given directly after explant preparation; 10 µL of NT-647-labeled rhCNTF (3 µg dose) was applied gently on top of each explant, followed by incubation for 24 h at 37 °C.

#### 2.5.3. Tissue Culture Fixation and Sectioning

##### Rat Retinal Explants

Treated rat explants were fixed in 4% PFA (Polysciences, Inc., Warrington, PA, USA) at 4 °C for 40 min. The fixative was then changed to 1% PFA and incubated at 4 °C overnight. Retinae were then washed with PBS for 10 min and cryoprotected by incubation in graded sucrose solutions (10%, 20%, and 30%). Subsequently, tissues were embedded in Tissue-Tek^®^ O.C.T.™ Compound (Science Services GmbH, Munich, Germany). Vertical sections (14 μm) were obtained on a Leica CM3050S Microtome (Leica Biosystems, Wetzlar, Germany), air-dried at 37 °C for 1 h, and stored at −20 °C until use.

##### Bovine Retinal Explants

Treated bovine explants were fixed by replacing the medium below the filter with 4% PFA (in PBS). After 2 h of incubation at 4 °C, PFA was discarded and replaced with 30% sucrose solution (in PBS) and incubated overnight at 4 °C. Explants were snap-frozen in Tissue-Tek^®^ O.C.T Compound using liquid nitrogen and sections (16 µm) cut from four different region of the explant with cryostat (Leica CM3050s).

#### 2.5.4. Culture Staining and Imaging

##### Rat Retinal Explants

To prepare tissue sections from retinal explants for imaging, frozen cryosections were first air-dried at 37 °C. Slides with fixed sections of retinal tissue were washed 3 times with PBS and then mounted in Vectashield^®^ (Vector Laboratories, Inc., Burlingame, CA, USA). Immunofluorescence staining of the mounted sections was carried out as described in the Supplement.

Mounted sections were imaged using a Zeiss Axio Imager Z1 ApoTome microscope equipped with a Zeiss AxioCam digital camera and AxioVision 4.7 software (all ZEISS). To observe the penetration of labeled rhCNTF, acquired images from multiple explant areas were assessed manually for the presence of fluorescence.

##### Bovine Retinal Explants

Sections from retinal explants were incubated for 1 h at room temperature in blocking solution (5% goat serum) followed by overnight incubation at 4 °C with rabbit anti-Collagen IV antibody (1:200) (Abcam plc., Cambridge, UK). Next, sections were stained with Alexa Fluor™ 488-labeled goat anti-rabbit secondary antibody (1:500) (Thermo Fisher Scientific) and 10 µg/mL Hoechst 33,342 (Invitrogen™, Thermo Fisher Scientific) for 1 h at room temperature.

Sections were mounted with Vectashield^®^ (Vector Laboratories) and prepared for imaging. Sections were imaged with a Leica TCS SP8 confocal microscope using 20x (HC PL APO 20x/0.75 IMM CORR CS2) and 93x (HC PL APO 93x/1.30 motCORR STED WHITE) objectives (all Leica Microsystems GmbH, Wetzlar, Germany). As before, the presence of fluorescence was assessed manually from multiple explant areas to observe penetration of labeled rhCNTF.

## 3. Results

### 3.1. Protein Production

**Figure 1:**
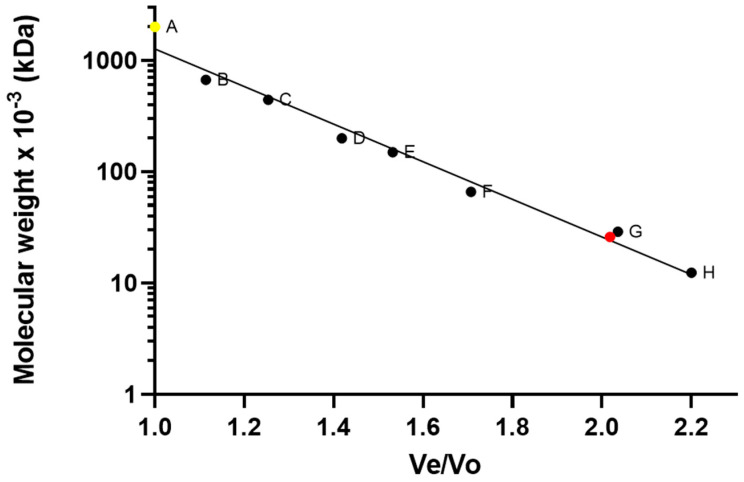
Size-exclusion chromatography of IMAC-purified recombinant human ciliary neurotrophic factor (rhCNTF). Pooled and concentrated eluates from IMAC batch-purification were subjected to SEC in a Superdex 200 prep grade packed C16/40 column with 100 mM NaH2PO4, 300 mM NaCl, pH 8.0, 1 mM DTT as the running buffer with a flow rate of 0.5 mL/min. The column was calibrated with blue dextran (A; 2000 kDa), thyroglobulin (B; 669 kDa), apoferritin (C; 443 kDa), β-amylase (D; 200 kDa), alcohol dehydrogenase (E; 150 kDa), bovine serum albumin (F; 66 kDa), carbonic anhydrase (G; 29 kDa), and cytochrome c (H; 12.4 kDa). rhCNTF eluted at 56 mL (shown in red), with an estimated molecular weight of 26 kDa.

After expression, harvesting, and lysis, expressed rhCNTF was found mostly in the lysate supernatant, indicating soluble protein overexpression. The soluble protein was purified from the lysate with immobilized metal-ion affinity chromatography (IMAC), and the eluted fractions analyzed with SDS-PAGE (not shown). Fractions containing rhCNTF were pooled, concentrated, and subjected to SEC purification, with rhCNTF eluting from the column as a mostly solitary major peak at 56 mL (Figure 1), corresponding to an estimated molecular weight of approximately 26 kDa and in accordance with published literature and earlier SDS-PAGE analysis [42], with void volume aggregates and smaller oligomers eluted as distinct minor peaks and readily discarded (data not shown). Four separate batches of rhCNTF were purified in high purity, on average yielding 70 mg of protein per liter of culture.

### 3.2. rhCNTF In Vitro Bioactivity

**Figure 2:**
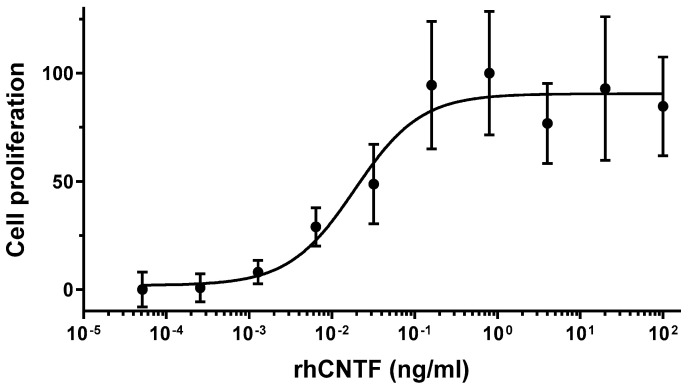
5-bromo-2’-deoxyuridine (BrdU) incorporation assay analysis of the in vitro biological activity of rhCNTF to support the proliferation of TF-1.CN5a.1 cells. rhCNTF supports the short-term proliferation of TF-1.CN5a.1 cells, with BrdU incorporation into synthesized DNA observed as a proxy for cell proliferation. Each value shown as average mean of three biological replicates ± SD. Data were normalized to highest bioactivity of rhCNTF.

Aside from previously confirmed binding to the cognate receptor CNTFRα [42], the in vitro activity of purified rhCNTF was assessed based on the proliferation of TF-1.CN5a.1. The cell-line is engineered to stably express CNTFRα, and CNTF-mediated stimulation can hence support the short-term proliferation of the cells. As illustrated in Figure 2, our purified rhCNTF supported the short-term proliferation of the cells, observed via BrdU incorporation, in a dose-dependent manner. An approximate EC_50_ of 19 pg/mL (0.8 pM) was estimated for this effect, indicating that our purified rhCNTF was biologically active.

### 3.3. Characterization and Stability of rhCNTF

#### 3.3.1. Circular Dichroism

**Figure 3:**
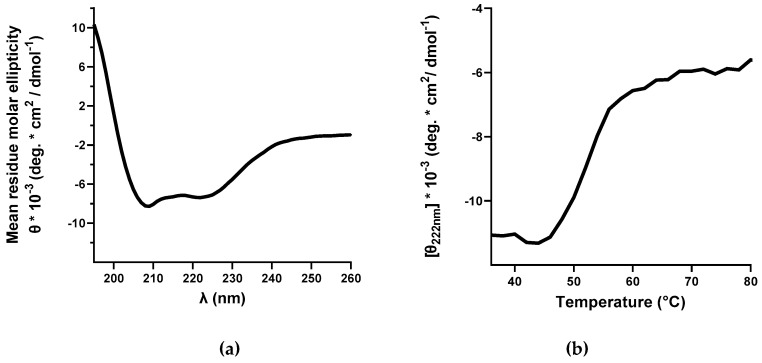
Far-UV circular dichroism analysis of rhCNTF. (**a**) Analysis of rhCNTF secondary structure at 25 °C: characteristic maximum around 195 nm with minima at 208 and 220 nm point to a highly helical protein, reflecting the correct α-helical secondary structure and folding of purified rhCNTF. (**b**) Thermal denaturation of rhCNTF observed as changes in ellipticity at 222 nm, reporting α-helical unfolding.

To investigate the secondary structure and folding of purified rhCNTF, far-UV CD analysis was carried out. In the recorded spectrum, an intense maximum around 190 nm with minima at 208 and 220 nm were observed (Figure 3A), spectral features corresponding to a highly α-helical secondary structure [54]. The high estimated percentage of helicity (>50%), as obtained by spectral deconvolution with BeStSel, along with further comparison with published results, confirm the correct secondary structure and folding of purified rhCNTF [10,55,56,57]. Furthermore, CD analysis of the thermal denaturation of rhCNTF yielded a T_onset_ of 46 °C and a T_m_ of 53 °C (Figure 3B), in accordance with published literature [58].

#### 3.3.2. ThermoFluor

First, a pre-screen was carried out to determine the optimal working concentrations of rhCNTF and SYPRO orange. The combination of 3.3 µM rhCNTF and 5X SYPRO Orange gave a maximal range of fluorescence and the best signal-to-noise ratio and was used for all subsequent measurements.

**Figure 4:**
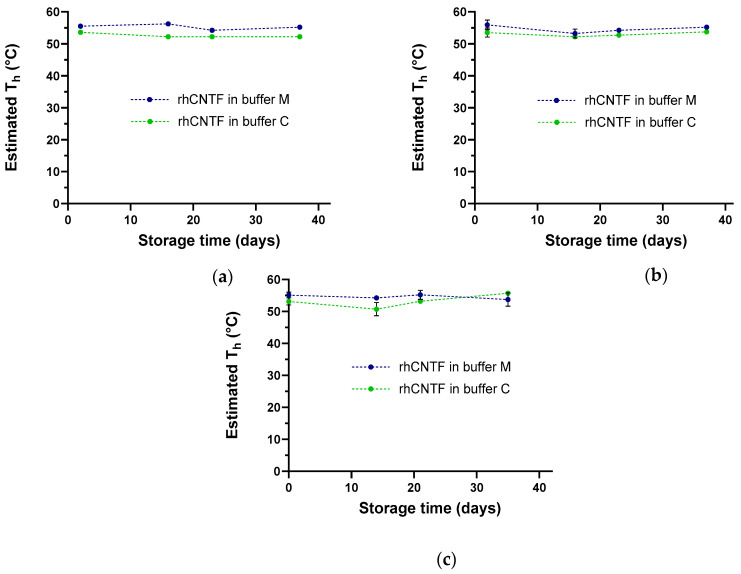
T_h_ estimation of rhCNTF during storage (**a**) on ice at 4 °C, (**b**) −80 °C, and (**c**) on ice at 4 °C post-thawing.

We screened 94 different buffers (Table S1) for optimal thermal stability of the purified rhCNTF. In general, poor thermal stability was observed at low pH, with highest thermal stability observed in slightly acidic to neutral pH; the presence of salt was observed to both increase and decrease thermal stability, depending strongly on the buffering component (Table S4). Two buffers yielding clear fluorescence responses and high T_h_ estimates were chosen for further use: 100 mM 2-(*N*-morpholino)ethanesulfonic acid (MES), 500 mM NaCl, pH 7.0 (buffer M), and 100 mM sodium citrate, pH 5.6 (buffer C); both buffers were supplemented with 1 mM dithiothreitol (DTT) to prevent covalent dimer formation via free cysteines in rhCNTF monomers. To gain insight on rhCNTF’s stability during storage, ThermoFluor measurements were carried out with rhCNTF stored on ice at 4 °C as well as at −80 °C in these buffers. As the observed T_h_ showed only minor changes during the study, this was taken to reflect the retained conformational stability of rhCNTF (Figure 4A–C). Furthermore, protein loss due to precipitation was not observed upon visual inspection of rhCNTF cryo-stored in these buffers (data not shown).

#### 3.3.3. Dynamic Light Scattering

Hydrodynamic size estimation of rhCNTF was carried out with DLS. The mean estimated R_h_ of rhCNTF was 3.42 ± 0.50 nm and 2.95 ± 0.22 nm for protein stored on ice at 4 °C in buffers M and C, respectively (4 × 3 measurements). This peak likely reflects both monomeric and dimeric rhCNTF, as DLS often cannot distinguish between species of such similar sizes [59]. This peak had a slightly higher and more variable polydispersity index (PdI) of 0.06 ± 0.04 with rhCNTF stored in buffer M, whereas when stored in buffer C, the same peak was associated with a low PdI of 0.03 ± 0.01.

**Figure 5:**
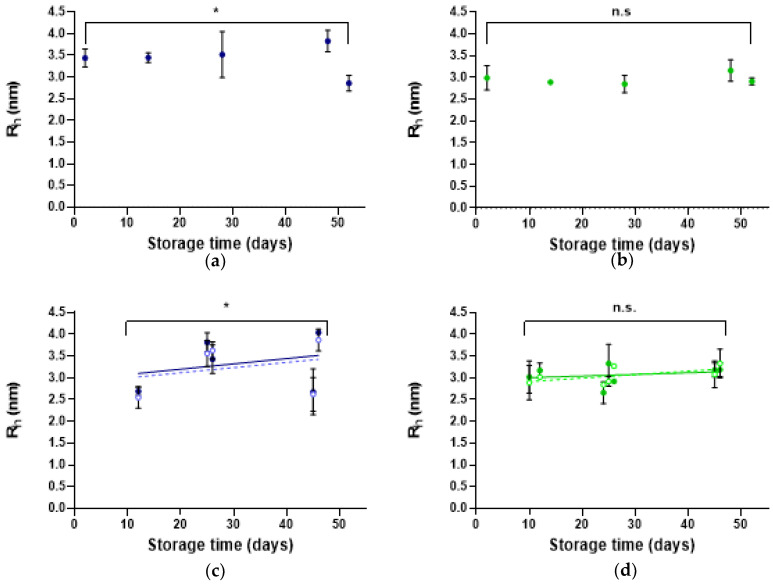
rhCNTF hydrodynamic radius estimates during storage on ice at 4 °C in (**a**) buffer M, and (**b**) buffer C, and at -80 °C in (**c**) buffer M, and (**d**) buffer C. Solid circles denote samples that were unmixed after thawing, and open circles denote centrifugally cleared thawed samples. Over time, more variations with the R_h_ of rhCNTF stored in buffer M were observed compared to only minor R_h_ variations with rhCNTF stored in buffer C. Each value shown as average mean ± SD (n = 3 technical replicates; one-way ANOVA, * *p* < 0.05, n.s. = no significance).

The size and oligomeric state of rhCNTF were monitored by following changes in the R_h_ of the monomer/dimer peak and volume distribution percentage of high molecular weight (HMW) species during storage on ice at 4 °C (Figure 5A,B and Table S5) as well as at −80 °C (Figure 5C,D and Table S6). To examine whether centrifugation affected sample quality and detecting HMW species, a comparison was made between thawed and (a) unmixed vs. (b) centrifuged samples (Figure 5C,D, Tables S5 and S6).

During storage at 4 °C, statistically significant differences were not observed in the (monomer/dimer) peak R_h_ of rhCNTF stored in buffer C (Figure 5B,D), whereas when stored in buffer M, statistically significant changes in the peak R_h_ were observed (Figure 5A,C). By intensity distribution, HMW species below, in, and occasionally above the sub-visible range (100–1000 nm) were detected (Table S5). However, as the scattering intensity is proportional to the sixth power of the particle radius, when expressed in intensity distribution, the observed intensities and population percentages are disproportionately biased towards the HMW species relative to dominant smaller particles [59]. When instead expressed in volume distribution, the contribution of HMW populations is significantly reduced. As observed from the volume distribution, HMW species were absent in buffer C, and as this remained unchanged (Table S5), and the protein was concluded not to cluster into aggregates during storage at 4 °C. When stored at −80 °C, detected aggregates were likewise negligible (less than 0.1% of the total) or completely absent, and as removal by centrifugation had no effect, rhCNTF does not undergo aggregation during nor due to cryo-storage (Table S6).

rhCNTF does, however, seem to undergo more aggregation in buffer M, as HMW particles were observed in both storage conditions also from volume distribution profiles/percentages, and as centrifugation was observed to influence the detection of aggregates (Tables S5 and S6).

**Figure 6:**
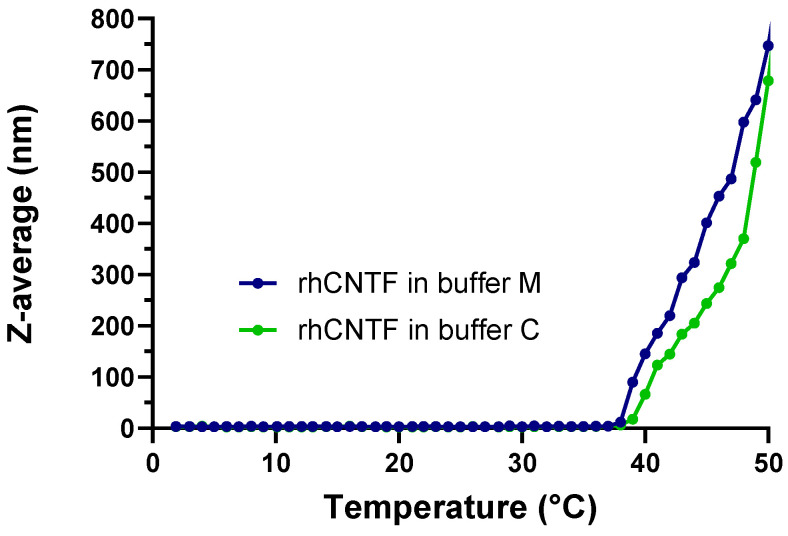
Thermal aggregation curve of freshly purified rhCNTF. Aggregation of purified rhCNTF was measured by following the estimated Z-average (nm) with dynamic light scattering (DLS) as a function of temperature. Appearance of high molecular weight (HMW) species is observed at temperatures above 38 °C in both buffers.

The onset of rhCNTF aggregation was studied during thermal ramping. Thermal aggregation of rhCNTF started at a T_agg_ of 38 °C in both buffers as determined with the proprietary multi-parameter analysis (Figure 6); in volume distribution analysis, HMW species appear at temperatures above 40 °C.

### 3.4. rhCNTF In Vivo Efficacy Study

#### 3.4.1. ERG: First Study Set

**Figure 7:**
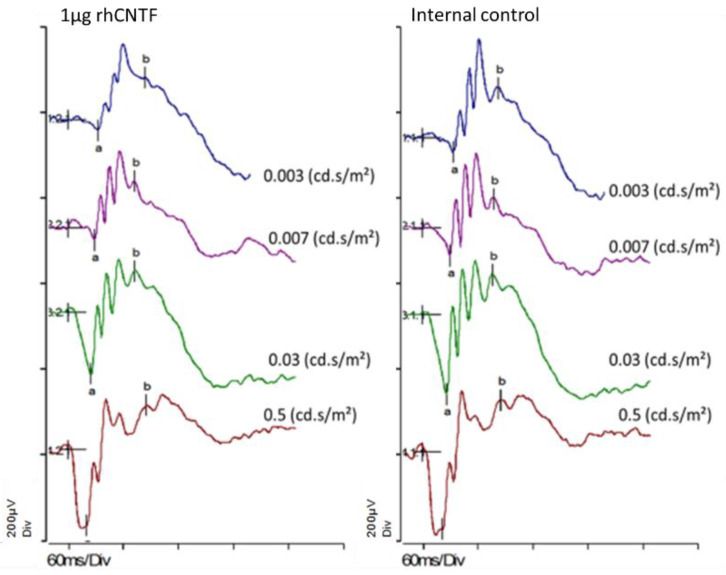
Scotopic Electroretinogram (ERG) recorded one week after 1 µg rhCNTF injection to the left eye. Equal visual acuity between the rhCNTF-treated eye and the contralateral untreated eye (internal control) was observed.

Figure 7 presents individual scotopic ERG results for an animal in the 1 µg rhCNTF dosage group, one-week post-injection. The α and β wave amplitudes between the treated eye and the untreated contralateral eye do not substantially differ. Furthermore, the similarity of the graphs indicates equal visual capacity of the 1 µg rhCNTF treated and the untreated eye.

**Figure 8:**
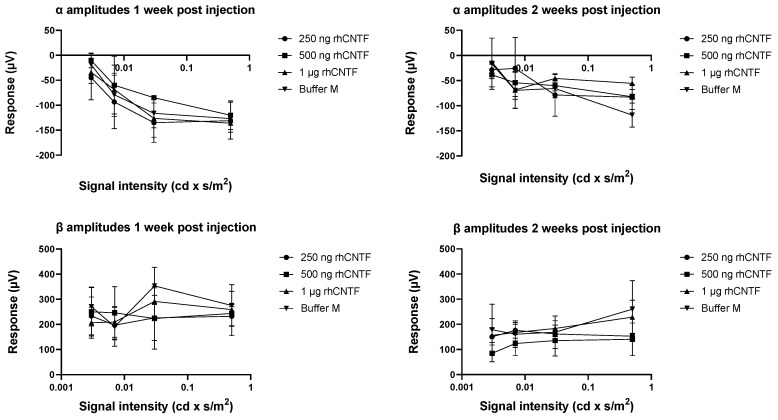
Scotopic α and β wave amplitudes one- and two-weeks post-injection. No statistically significant difference in the responses between any of the treatment groups could be detected.

**Figure 9:**
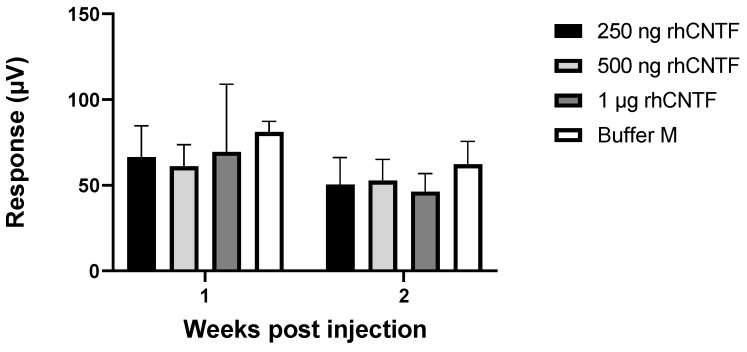
Photopic beta wave amplitudes at stimulus intensity 1 cd × s/m^2^ one- and two-weeks post-injection. No statistically significant difference in the responses between any of the treatment groups at different timepoints could be detected.

Figure 8 presents the recorded scotopic α and β wave amplitudes in different treatment groups one- and two-weeks post-injection (n = 3). Figure 9 presents the recorded photopic β wave value distribution at flash stimulus 1 cd × s/m^2^ (n = 3). No statistically significant differences could be seen in the scotopic α and β wave values or photopic β wave values between the treated and untreated eyes in any treatment group at any flash intensity or timepoint (related samples Wilcoxon signed ranks test, significance level: *p* < 0.05). The differences in the parameter distribution across the different treatment groups were not statistically significant at any flash intensity in the scotopic or photopic ERG recorded 1 week or 2 weeks post-injection (Kruskal–Wallis test, significance level: *p* < 0.05). The large range in the observed values indicates a large within-group variation in the test population.

#### 3.4.2. ERG: Second Study Set

**Figure 10:**
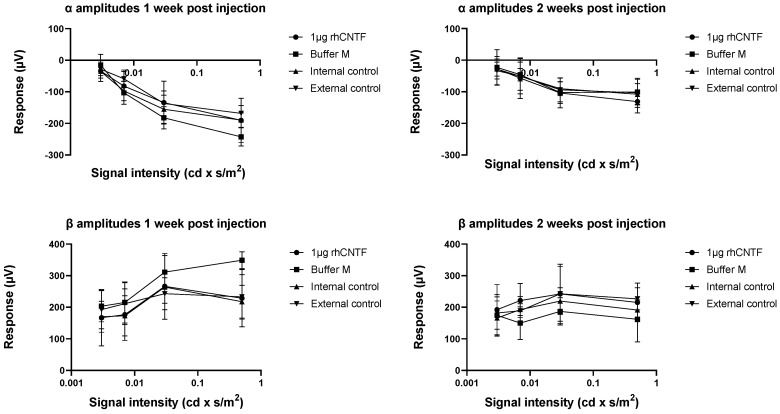
Mean and SD of scotopic alpha and beta wave amplitudes one and two-weeks post-injection. Scotopic 0.5 cd × s/m^2^ β wave amplitudes recorded one-week post-injection were significantly higher in the 2-(*N*-morpholino)ethanesulfonic acid (MES)-treated group than in 1 µg rhCNTF-treated group (Kruskal–Wallis test, *p* = 0.018) and NControl group (*p* = 0.025). However, the 1 µg CNTF-treated group and the NControl group did not show any statistically significant difference between their mean values (*p* = 0.831) (n = 6).

**Figure 11:**
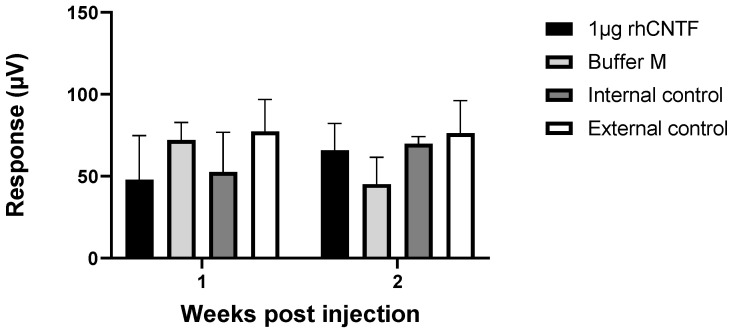
Mean and SD of photopic beta wave amplitudes at stimulus intensity 1 cd × s/m^2^ one and two-weeks post-injection. No statistically significant difference in the responses between any of the treatment groups at different time points could be detected (n = 6).

Mean values and standard error of the scotopic α and β wave amplitudes recorded one- and two-weeks post-injection are presented in Figure 10 (n = 6). Figure 11 presents the recorded photopic β wave value distribution at flash stimulus 1 cd × s/m^2^ (n = 6). Only in the highest scotopic ERG stimulus intensity recorded two weeks post-injection, a statistically significantly steeper α wave was observed in the 1 µg rhCNTF-treated eyes than in the contralateral untreated eyes, used as internal control (related samples Wilcoxon signed ranks test, significance level: *p* < 0.05, *p* = 0.043). No statistically significant differences could be seen in the scotopic α and β wave values or photopic β wave values in other stimulus intensities or timepoints between the 1 µg rhCNTF-treated and internal control eyes. Comparison of the recorded scotopic α and β wave amplitudes in the 1 µg rhCNTF-treated eyes between the two time points showed no significant change at any flash intensity, indicating no change in visual acuity over time (Figures S1 and S2) (related samples Wilcoxon signed ranks test, significance level: *p* < 0.05). A comparison of parameter distribution between the treatment groups showed no statistically significant differences in parameter distribution between the treated and control eyes (Figures S3–S5) (Kruskal–Wallis test, significance level: *p* < 0.05).

#### 3.4.3. Histology

**Figure 12:**
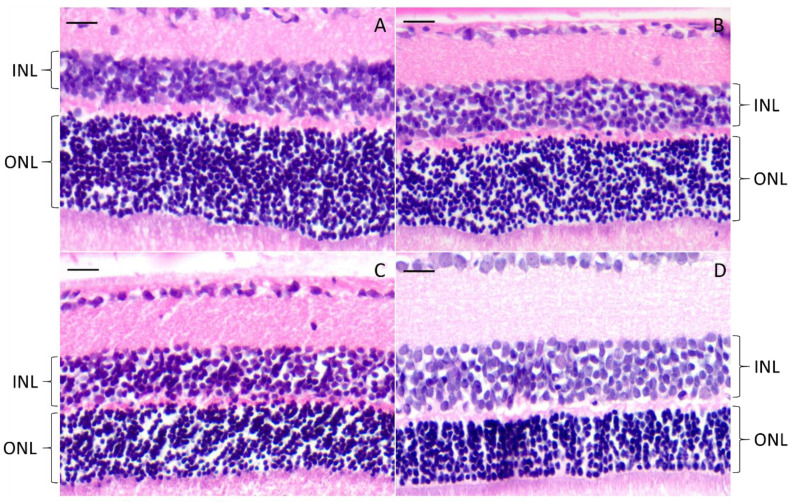
Effect of rhCNTF treatment on the morphology of the retina. Histological analysis of retinal sections did not show clear benefit of treatment with 1 µg rhCNTF (**a**) compared to internal control (**b**), Buffer M control (**c**), and Ncontrol (**d**). Scale bar 20 µm. INL, inner nuclear layer; ONL, outer nuclear layer.

The effect of rhCNTF treatment on the morphology of the animals in the second study set was evaluated by the thickness and density of the outer nuclear layer (ONL) of the retina. Representative histological samples prepared of MES (n = 2) NControl (n = 3) and 1 µg CNTF (n = 4) treated animals are presented in Figure 12. One animal in the 1 µg CNTF-treated group seemed to have denser and thicker ONL in the treated eye (A) than in the contralateral eye (B), however, this effect could not be seen in any other animals of the same treatment group. There was also variation in the progress of photoreceptor degeneration between individual animals at the same time-point.

### 3.5. Retinal Penetration of Labeled rhCNTF

In vivo, rhCNTF did not show any observable effect on photoreceptor function nor survival. Since this raised concerns on whether the IVT injected protein permeates the retina to reach CNTF-responsive target cells, this was studied with fluorescently labeled rhCNTF in an ex vivo setting with retinal explants.

**Figure 13:**
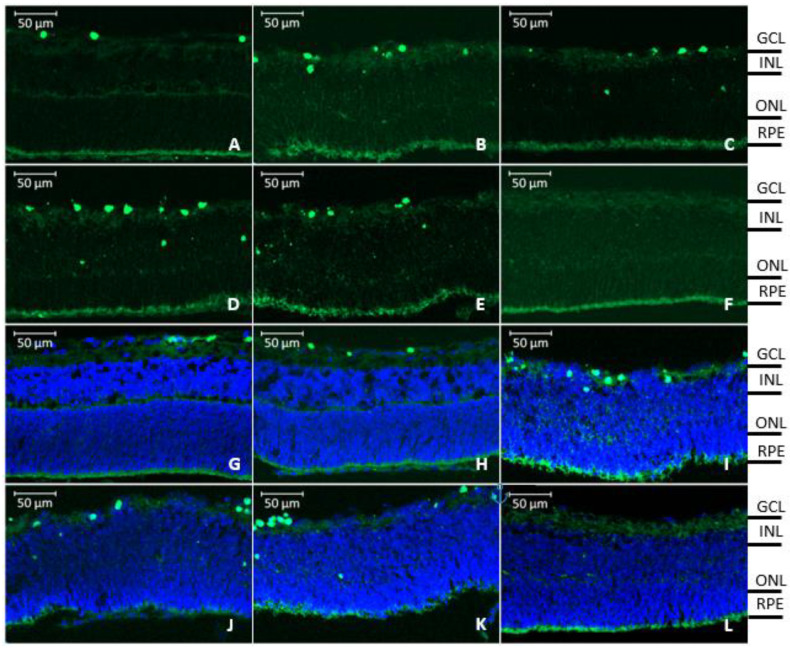
rhCNTF penetration in rat retinal explants as visualized without (**A**–**F**) and with DAPI counterstaining (**G**–**L**). Alexa Fluor™ 488 labeled rhCNTF (green) penetrates and distributes in the retina after apical administration, with rhCNTF-positive cells observed in the neural retina in layers ranging from the GCL to the INL (**A**–**E**,**G**–**K**). No rhCNTF penetration is seen with basolaterally applied protein (**F**,**L**). GCL, ganglion cell layer; INL, inner nuclear layer; ONL, outer nuclear layer; RPE, retinal pigment epithelium.

In the rat retinal explants, fluorescence from labeled rhCNTF was observed mainly in the ganglion cell layer (GCL) but also deeper, for example in the inner nuclear layer (INL), 24 h after apical application, as illustrated in Figure 13A–E,G–K. No evident fluorescence from labeled rhCNTF was observed 24 h after basolateral application, as shown in Figure 13F,L.

**Figure 14:**
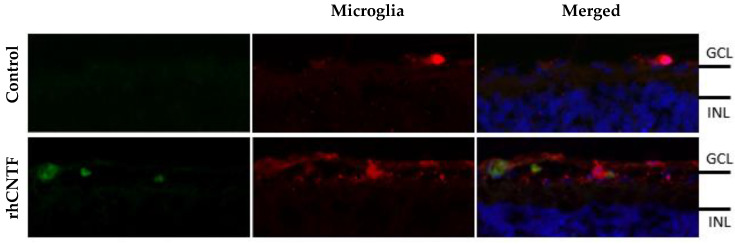
rhCNTF co-localizes with microglia in the ganglion cell layer. Fluorescence from Alexa Fluor™ 488 labeled rhCNTF (green) co-localizes with Iba-1 immunopositivity (red). GCL, ganglion cell layer; INL, inner nuclear layer.

Since fluorescence from labeled rhCNTF was observed mostly in the GCL, and as retinal cells expressing CNTFRα include e.g., retinal ganglion cells and Müller glia in this layer, staining to visualize Iba-1 immunoreactivity was carried out to identify microglial cells. Upon closer inspection, rhCNTF was observed to co-localize with microglia in the rat explants, as illustrated in Figure 14.

**Figure 15:**
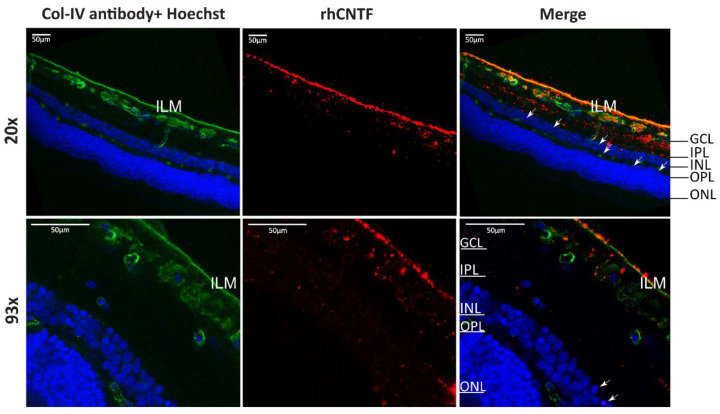
rhCNTF penetration in bovine retinal explant. NT-647 labeled rhCNTF (red) readily penetrates across inner limiting membrane (ILM) and distributes in the retina after apical administration, with evident fluorescence observed in the neural retina in layers ranging from ganglion cell layer (GCL) to outer plexiform layer (OPL). ILM, inner limiting membrane; GCL, ganglion cell layer; IPL, Inner plexiform layer; INL, inner nuclear layer; OPL, outer plexiform layer; ONL, outer nuclear layer.

24 h after apical application, fluorescence from the labeled rhCNTF was observed in most retinal layers in the bovine retinal explants, as shown in Figure 15 and Figure S6. Whereas fluorescence can be observed mostly at the ILM and GCL, rhCNTF-positivity is also observed in the outer plexiform layer (OPL) and the ONL.

## 4. Discussion

Pharmacotherapeutic intervention affecting the disease mechanisms and causes is often limited to specific pathologies. For example, using anti-VEGF biologicals is restricted to treating posterior eye segment conditions featuring pathological neovascularization. Instead of blocking pathways ultimately leading to neurodegeneration, directly supporting the function and survival of neurons—for example with different neurotrophic factors—is an attractive strategy to alter the course of pathology, and a potentially more universal approach in treating neurodegenerative diseases of diverse etiologies. The cytokine CNTF is of great interest as the protein’s neuroprotective effects on various retinal neurons have been demonstrated in numerous retinal disease models, subsequently ushering clinical evaluation in treating various retinal conditions in humans. 

In vitro bioactivity in a cell-based assay was used to verify the biological activity of purified rhCNTF by assessing its effects on the proliferation of a CNTF-responsive cell line. It is known that the lymphoblastoid cell line TF-1 responds to multiple cytokines, e.g., GM-CSF, IL-3, and erythropoietin by proliferating [60]. As TF-1 cells express IL-6Rα, another receptor subunit for CTNF [11], they can respond to exogenous CNTF. However, this binding is of markedly lower affinity compared to that with CNTFRα, resulting in a less pronounced proliferative response; simultaneous addition of soluble CNTFRα makes the TF-1 considerably more responsive to exogenous CNTF [61]. To circumvent the need to add exogenous CNTFRα to cultured TF-1 cells, we used TF-1.CN5a.1, a TF-1 derived cell line transfected to stably express CNTFRα. The cells are thus capable of proliferating in response to much lower CNTF concentrations, and the cell line can be used to assess, e.g., the activity and potency of CNTF and its variants. Here, we demonstrated that aside from binding to its cognate receptor, our rhCNTF has the correct α-helical structure (Figure 3A) and is biologically active as it supported the short-term proliferation of TF-1.CN5a.1 cells with an estimated EC_50_ of 19 pg/mL (0.8 pM).

### 4.1. Buffer Screening and Stability Studies

We investigated the stability of rhCNTF in various buffers. Although widely used in formulations for therapeutic proteins, sodium phosphate buffer has been reported to be unsuitable for the cryo-storage of many proteins, as the buffer undergoes so-called freeze acidification [62,63]. Particularly, the dibasic phosphate salt undergoes a selective crystallization process during cooling, leading to a decrease of up to 3 pH units during freezing [62,64]. Furthermore, low temperature, freeze-concentration of solutes, and the formation of ice-aqueous interfaces are but some examples of the physicochemical changes and potentially destabilizing stresses that proteins may experience during freezing [62]. Destabilization of the protein structure may lead to (partial) denaturation of the protein structure, and further to protein aggregation. Indeed, we observed major loss of purified protein when rhCNTF was stored in a sodium phosphate buffer at -80 °C. Thus, a more suitable buffer was required for the protein.

Here, different buffer, salt, and pH conditions were screened with ThermoFluor to assess their effects on the conformational stability and T_h_ of rhCNTF. Although high T_h_ estimates were seen with several buffers, the observed fluorescence responses were low and the negative peaks in the first derivative plots were poorly discernible with certain buffers and the T_h_ was therefore challenging to estimate reliably. Among the screened buffers, two with clear fluorescence responses and high T_h_ estimates reflecting high thermal stability of rhCNTF (100 mM MES, 500 mM NaCl, pH 7.0, and 100 mM sodium citrate, pH 5.6; both supplemented with 1 mM DTT) were chosen and further tested. Although the number of screened conditions is arguably limited, and as replications could admittedly reinforce our confidence in the obtained results, they, along with the DLS results, nonetheless provide information on suitable buffers that can serve as a basis of further formulation development for rhCNTF. Moreover, our results indicate useful starting points around which ‘fine’ screening, i.e., buffer optimization with different additives (e.g., glycerol, surfactants, polyols, and reducing agents) is advisable to further increase the stability of rhCNTF [65]. As the T_h_ estimates remained fairly consistent during the follow-up measurements (Figure 4), despite being insufficient for rigorous statistical analysis, this led us to draw the conclusion that the conformational stability of our rhCNTF was retained during the study and in our experimental setup.

Although thermal aggregation of rhCNTF initiates at temperatures above 38 °C in both buffers, this is not concomitant with heat-induced unfolding. As measured with CD and ThermoFluor, rhCNTF starts to unfold at temperatures above 46 °C (T_onset_; Figure 3B). The onset of aggregation at 38 °C is, however, not necessarily due to association via exposed hydrophobic patches, as can be expected to occur later at higher temperatures. Further elucidation is warranted to gain a more comprehensive understanding of the protein’s aggregation behavior. Moreover, upon administration, the protein gets exposed to the vastly different, complex, and potentially hostile in vivo environment that has been postulated to influence the formation and nature of aggregates [66,67]. Therefore, as the T_agg_ is close to normal body temperature, it cannot be ruled out that rhCNTF could not undergo aggregation to some degree in oculo after administration at 35 °C in the vitreous. Although engineered CNTF variants with improved chemical and physical properties were originally developed for CNS delivery [41], it would be of interest to investigate whether they are more stable in the eye as well.

Particle sizing results suggest that oligomerization of rhCNTF differs in the two buffers. Overall, the slightly larger R_h_ estimate with rhCNTF in buffer M is likely due to the composition of the solvent. The presence of high salt concentration – in this case, 500 mM NaCl – can shield the charge-mediated protein-protein repulsive forces, decreasing the diffusivity of macromolecules in solution and appearing as an increase in measured hydrodynamic size [68,69]. When stored in this buffer, appearance of HMW species is detectable, whereas no dramatic oligomerization takes place in buffer C in either storage condition (Tables S3 and S4). In intensity distributions, HMW particles were detected in both buffers, but when expressed in volume distribution, larger particles were evident only in buffer M (Table S4). Although based on volume distribution profiles, the amount of HMW species is negligible in buffer C; further development of a therapeutic rhCNTF lead molecule and its formulation may still require additional engineering and optimization to prevent the formation of even trace aggregates detected only in intensity distributions here.

Even though DLS is a powerful method for the detection and rate determination of oligomer and larger aggregate formation, the underlying mechanism(s) of aggregation cannot be determined with this method. Our studies were not set up for uncovering whether trace oligomers and HMW aggregates present in storage and induced during thermal ramping are irreversible, covalent, insoluble, etc., and this would require further studies with orthogonal methods, such as SEC with multi-angle laser light scattering (MALLS), analytical ultracentrifugation (AUC), and nanoparticle tracking analysis (NTA) [70,71]. Such studies could also yield results to corroborate the findings presented here. Lastly, although not feasible during our study, we realize that assessing rhCNTF’s binding to CNTFRα and/or effect on cell proliferation during our stability studies could provide even more concrete proof of the stability and retention of rhCNTF function during storage.

Finally, the intraocular stability of therapeutic proteins is of immense importance; even though the eye is often considered an immune-privileged site, it is possible that therapeutic protein aggregates in the vitreous would induce immune responses [67] that could affect the efficacy and safety of these drugs. Studies on therapeutic protein stability, aggregation, and immunogenicity after intravitreal administration are still, however, scarce. Although not studied here, in situ stability concerns are equally pertinent with IVT CNTF, warranting further investigations that could, for example, be carried out by adopting recently described ex vivo approaches [72,73] to study these aspects with rhCNTF.

### 4.2. In Vivo Bioactivity of rhCNTF

Based on the results from the first study set, the rhCNTF treatment did not have a dose-dependent positive or negative effect on the recorded ERG values, and the photoreceptors in the rhCNTF-treated eyes did not seem to benefit from the treatment compared to the control eyes. As there was no significant reduction in the photoreceptor function over time, it is possible that the photoreceptor degeneration did not progress dramatically between the recordings, or that the photoreceptor degeneration was already rather extensive in the animals aged 28 days, when the ERG was recorded for the first time. Therefore, in the second study set, the animals were treated at an earlier time point, around the onset of photoreceptor degeneration. In the second study set, the group treated with 1 µg rhCNTF was the only group that showed no significant reduction in the scotopic ERG values over time; however, this finding alone was not substantial enough to prove any therapeutic effect of CNTF treatment on photoreceptor degeneration.

While ERG has proven to be an effective method in evaluation of retinal function in RCS rats, it appears that the reduction of the ERG response might not always correlate well with the anatomical loss of photoreceptors [74]. There has also been some scientific debate whether CNTF is, in fact, detrimental to the rod photoreceptors as in some in vivo studies intraocular CNTF therapy has paradoxically reduced the scotopic ERG responses. A dose-dependent transient reduction in ERG responses has been observed in normal Long-Evans rats after IVT injection of CNTF that corresponded with a morphological reduction in ROS length with full recovery observed by three weeks post-injection [75]. Multiple teams using viral-mediated gene therapy have also reported diminished ERG amplitudes in treatment groups or no observable improvement in ERG, whilst the number of preserved photoreceptors has been higher in treatment groups [18,31,32,76]. Some teams, however, have been able to improve the scotopic or photopic ERG values with viral CNTF treatments or CNTF secreting devices in different animal models [33,35,36,40]. There is evidence that CNTF can reduce the rhodopsin levels and other proteins important in the phototransduction cascade [25]. Therefore, the photoreceptor preservative effect of CNTF could be counteracted by the negative effect it presents on the functionality of the preserved photoreceptor in the retina. However, in our experiments, rhCNTF did not affect the retinal morphology, nor the function.

**Figure 16:**
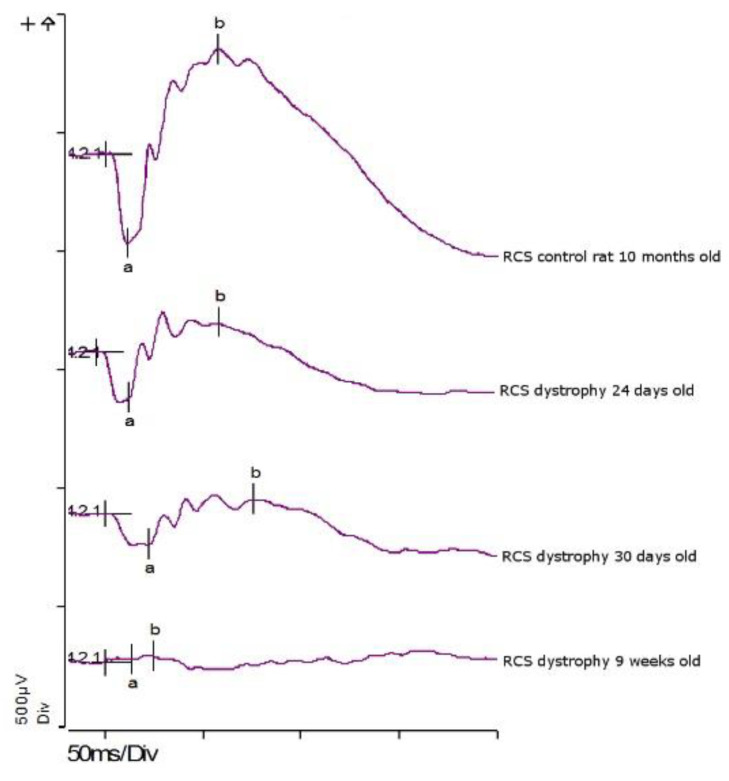
Scotopic 0.5 cd x s/m^2^ ERG of Royal College of Surgeons (RCS) control animal at the age of 10 months, RCS dystrophic rat at the age of 24 days, 30 days, and 9 weeks. The progressive photoreceptor degeneration and loss of photoreceptor functionality can be seen as the α and β wave (marked as a and b in the graph) amplitudes decrease in magnitude rather rapidly between 24 and 30 days in the RCS dystrophic rat, whilst the 10-month old RCS control animal exhibits distinct α and β wave responses. The fully blind nine-week-old RCS dystrophy rat has no measurable α or β wave amplitudes.

RCS rat has a mutation in the gene encoding tyrosine kinase receptor Mer, affecting the ability of RPE cells to phagocytise rod outer segment (ROS) debris [74,77,78]. With time, the ROS debris accumulates, eventually forming a layer between the RPE cells and photoreceptors, leading to gradual photoreceptor degeneration and full blindness by the age of two to three months [74]. The gradual decrease in the scotopic ERG responses in RCS rats is presented in Figure 16. Even though CNTF related photoreceptor preservative effect has been observed in RCS rat [14,30,33], it is possible that halting the rapid progression of photoreceptor generation in this animal model is challenging with a single bolus injection of therapeutic protein, making sustained delivery systems more feasible options.

The pharmacokinetics (PK) of CNTF have so far been studied in rats only with systemic CNTF, reporting a short systemic half-life of 2.9 min; when discussing the ocular PK of CNTF, the 1994 papers by Dittrich et al. [79] and Sendtner et al. [80] and subsequent publications citing these are commonly referred to. Although the early papers discuss the PK of systemically administered CNTF in rats, in the citing literature the results are often straightforwardly extrapolated to represent the in vivo PK of CNTF in general, and it has been assumed that CNTF would lose its therapeutic effect quickly also in the vitreous after IVT injection due to rapid clearance [36,81]. Regrettably, claiming the ocular half-life of CNTF to be in the same time-scale—of mere minutes—as in the circulation, is a gross and lamentable misinterpretation of the publications’ results.

Studies on the intravitreal pharmacokinetics of macromolecules carried out in rabbits have shown that macromolecules can have a half-life of several days, and that the half-life of the macromolecule in the vitreous is greatly dependent on the intravitreal clearance, which in turn is small for those macromolecules that cannot pass the BRB, as is thought to be the case with CNTF [6,82]. Biologicals are eliminated from the vitreous predominantly via the anterior route [83], with the hydrodynamic size considered as the major determinant in the ocular half-life of a given biological [84,85]. Although experimental data on the vitreal half-life of CNTF is as of writing scarcely published, in a study with rat CNTF injected in mouse vitreous—although owing to practical limitations the vitreal CNTF levels were not assessed with total retinal levels reported instead [17]—we doubt that the levels would be as high as reported if the protein truly had a rapid vitreal elimination. Based on hydrodynamic radius estimations presented here, the calculated intravitreal half-life of rhCNTF are 4.67 days (R_h_ 2.95 nm) and 4.96 days (R_h_ 3.32 nm) (see Supplement), estimates that do not differ drastically from experimentally determined half-lives of other IVT administered macromolecules [86,87,88] and definitely not in the order of minutes. Even so, the vitreal half-life of CNTF is nevertheless finite and sustained intraocular release, and retinal delivery of CNTF has been explored utilizing various strategies: for example, an intravitreally injected hydrogel [58], cell-based systems [89], and several gene therapy approaches [18,90,91,92,93,94]. The most clinically advanced, Renexus^®^ (NT-501; Neurotech Pharmaceuticals, Inc., Cumberland, RI, USA) is an implant with encapsulated, genetically engineered ARPE-19 cells, that secrete and maintain low concentrations of human CNTF in the vitreous over several years [19]. Its neuroprotective effects have been evaluated in patients with dry AMD [95,96], CNGB3-achromatopsia [97], glaucoma [98], retinitis pigmentosa [5,19,99], and macular telangiectasia type 2 (MacTel 2) [100]. No efficacy was evident in CNGB3-achromatopsia patients [97], and only moderate efficacy was observed in RP patients and patients with geographic atrophy secondary to dry AMD [95,96]. However, after some therapeutic efficacy was observed in a recent phase 2 study in MacTel 2 patients [100], two phase 3 studies are now recruiting patients for evaluating the safety and efficacy of Renexus^®^ in treating MacTel 2 (NCT03316300, NCT03319849), whereas the results of a recent phase 2 study in glaucoma patients (NCT02862938) are currently expected.

### 4.3. Retinal Penetration and Effects of rhCNTF

As no apparent neuroprotective effects were observed with IVT administered rhCNTF in vivo, in case this was related to the protein’s ability to permeate into the retina, the postulated target cells were evaluated. Here, we demonstrate that after apical administration, our labeled rhCNTF—a small (26 kDa) protein—permeates into the neural retina in both explant models in 24 h. Consistent with findings with intravitreally administered antibody fragments [101], crossing the RPE was not observed with basolaterally applied rhCNTF as the protein did not permeate to the neural retina.

Given that the half-lives of intravitreally administered protein therapeutics are in the range of several days [102], we realize that in this context, the time scale of the given rhCNTF treatments is short. However, we chose to limit the treatments to 24 h in order to ensure the structural and physiological integrity of the explants, and on the other hand, that the stability of the protein–fluorophore conjugates was also retained during the experiments. Whilst not studied with the rat explants, and although based on collagen staining, the ILM appears intact in our experiments with the bovine explant (Figure 15 and Figure S6), adapting a recently described bovine explant model developed to ensure the integrity of the ILM and vitreoretinal interface [53] could be used to complement our findings, and to further define the contribution of these primary barriers in the retinal permeation of biologicals in general. It should also be noted that as the ILM in the rodent eye is poorly representative of human physiology compared to larger species, information obtained with bovine models may better reflect the in vivo situation in the human eye [53,101]. Even though the ILM is considered the primary barrier to retinal entry from the vitreous [53], e.g., the size-cutoffs remain undetermined [83], whereas the porosity of the ILM meshwork in human and animal eyes is still not fully clear [103]. Likewise, how recognized species differences relate to the applicability of different animals’ eyes in evaluating the retinal penetration of vastly different particles remains to be comprehensively elucidated. Whilst reports on the retinal permeation of biomacromolecules is by and large qualitative with sparse truly quantitative estimates published [83], as several full-sized mAbs [86,87,88] and even large particles such as nanocarriers have nonetheless been reported to penetrate into the retina [103], the ILM could, in fact, be an insignificant barrier to the retinal entry of biologicals, and in particular small proteins as seen with rhCNTF here.

As our retinal penetration experiments were not set up for studying the neuroprotective effects of our rhCNTF, conclusions thereof cannot be drawn here. Nonetheless, as labeled rhCNTF penetrated the ILM and permeated to retinal layers with CNTFRα-expressing cells, our results indicate that CNTF’s availability to the target cells should not be a limitation for its direct effects in the retina. Although published results suggest that the retinal microglia respond to CNTF in rats [23] and our labeled rhCNTF did indeed co-localize with local microglia in the GCL, it is not clear if these cells express CNTFRα and whether they are responsive to CNTF directly or indirectly. Therefore, verifying the microglial expression of the cognate receptor is pertinent to further corroborate our findings. The experiments could straightforwardly be expanded to assess the activation of the identified downstream signaling pathways of CNTF, such as JAK/STAT3, ras-MAPK, and PI3K/AKT [11,23]. Similarly, since the neurotrophic effects of CNTF are in part mediated by glial cells in the eye, mass spectrometry for example could be utilized to analyze microglial expression and secretion of other growth factors in response to stimulation with rhCNTF. Lastly, application of conditioned medium of microglial cells treated with rhCNTF to retinal explants and explant models of retinal degeneration and pathologies may also be carried out to assess e.g., protection of photoreceptors as well as to investigate the stimulation and secretory responses of Müller glia.

Although the bovine explants are viable only for a short duration [53], rodent explants can in comparison be maintained in culture for several weeks; explants exhibiting retinal pathologies and degeneration can also be induced, or, as eyes from preclinical animal disease models are available, prepared thereof [51,104]. As aging and diseases can affect the structures and the integrity of drug delivery barriers such as the BRB and the ILM [83,105], we see such rodent explant models potentially applicable in comparing how such changes in the retinal physiology influence the retinal permeation and distribution of protein therapeutics. In order to ensure that meaningful information is extracted in such studies, it is obviously essential to confirm that the explants retain their physiological integrity and truthfully reflect the physiological situation [106].

Even though the relevance of the retinal permeation of protein therapeutics is underlined in several publications [88,103,107,108], it is still an incompletely understood area of the intravitreal pharmacokinetics thereof. Aside from presented studies with rhCNTF, we envision the retinal explant models utilized in studying the retinal penetration and distribution of various other proteins of therapeutic interest, e.g., mAbs, Fab fragments, and Fc-fusion proteins. In particular, the explants could prove invaluable in systematically elucidating how different factors, such as protein hydrodynamic radius, charge, and FcRn-binding act as determinants in this regard. As it is possible that protein therapeutics with differing characteristics permeate into the retina at different rates, study designs naturally require robustness. On one hand, it has been proposed that retinal penetration might not be a universal requisite with all modalities [83]. On the other hand, there is support for opposing views; although current anti-VEGF therapeutics have extracellular targets, their permeation into the retina to bind free retinal VEGF is necessary for maximal VEGF inhibition based on a recent in silico study [109]. Even though posterior clearance is considered to play only a minor role in the vitreal clearance of protein therapeutics, it is indeed unclear whether penetrating into the retina interplays with the pharmacological effects of protein therapeutics in vivo. It is thus of great importance to investigate how retinal permeation of proteins, especially with different modes of action, is connected to their therapeutic efficacies in the eye and whether this can be influenced by protein engineering for example. Such studies could potentially provide explanations on why many proteins developed for ocular conditions have failed in clinical trials. Ultimately, they might offer valuable clues on overcoming recognized ocular barriers and thus help in bringing novel treatments to the clinic. Here, utilizing both in vivo as well as ex vivo methods, such as the described retinal explants, are essential [105].

## 5. Conclusions

Here, we focused first on verifying the bioactivity of rhCNTF, and subsequently on the characterization and stability studies of the protein. Whereas the protein’s activity was demonstrated in an in vitro cell proliferation assay, screening identified buffers in which the physicochemical stability of rhCNTF was retained during storage, providing a basis for further rhCNTF development and formulation efforts. Even though here rhCNTF showed no photoreceptor preservative effects in vivo, using ex vivo organotypic retinal explants we demonstrated, to the best of our knowledge, for the first time the permeation of labeled rhCNTF to the neural retina and additionally the exogenous cytokine’s co-localization with outer retinal macrophages. Whilst the lack of in vivo effects could, for example, be due to the extent of degeneration in the used disease model or the limited duration of action achievable with a single bolus injection, as the cytokine was observed to distribute to the neural retina, we conclude that this was not due to rhCNTF failing to reach responsive cells.

As the factors affecting retinal penetration of protein therapeutics are still unclear, there is a need for methods with which such information can be acquired. To improve the knowledge in this area, further studies are clearly necessary. As such, complementing the in vivo and in silico work carried out and described in recent literature, we hope our findings give impetus to—among other applications—explore the further use of retinal explant models to systematically elucidate the retinal permeation of protein therapeutics.

## Supplementary Materials

The following are available online at https://www.mdpi.com/1999-4923/12/7/611/s1, Figure S1: Mean and SD of recorded scotopic α and β wave amplitudes in the 2nd study set 1 week after intravitreal injection; Figure S2: Mean and SD of recorded scotopic α and β wave amplitudes in the 2nd study set 2 weeks after intravitreal injection; Figure S3: Left eye α wave distribution 1 week post-injection; Figure S4: Left eye β wave distribution 1 week post-injection; Figure S5: Left eye β wave distribution 2 weeks post-injection; Figure S6: rhCNTF penetration in bovine retinal explant; Table S1: Layout of the ThermoFluor buffer, salt, and pH screen; Table S2: Tissue processing procedure; Table S3: H&E staining protocol; Table S4: Heat map of rhCNTF T_h_ measured in ThermoFluor screen; Table S5: R_h_ estimation of rhCNTF stored on ice at 4 °C; Table S6: R_h_ estimation of rhCNTF stored at −80 °C.
