# Supplementary material for: Characterization, Stability, and In Vivo Efficacy Studies of Recombinant Human CNTF and Its Permeation into the Neural Retina in Ex Vivo Organotypic Retinal Explant Culture Models
Source: Pharmaceutics. 2020 Jun 30;12(7):611. doi: 10.3390/pharmaceutics12070611 (PMC7408322; doi:10.3390/pharmaceutics12070611)
Supplement: Supplementary file 1 [file pharmaceutics-12-00611-s001.pdf]

pharmaceutics

MDPI

Supplementary Materials: Characterization, Stability, and in Vivo Efficacy Studies of Recombinant Human CNTF and its Permeation into the Neural Retina in ex Vivo Organotypic Retinal Explant Culture Models

Jaakko Itkonen, Ada Annala, Shirin Tavakoli, Blanca Arango-Gonzalez, Marius Ueffing, Elisa Toropainen, Marika Ruponen, Marco G Casteleijn, and Arto Urtti

Protein expression and purification

For rhCNTF protein production, Rosetta 2(DE3)pLysS (Novagen) *E. coli* cells were transformed with expression plasmid pOPINF-hCNTF and the expression carried out as described earlier (Itkonen *et al.*, 2014). The subsequent purification of soluble rhCNTF from clarified cell lysates was carried out under native conditions by batch-mode immobilized metal-ion affinity chromatography (IMAC) with Protino® nickel iminodiacetic acid (Ni-IDA) resin (Macherey Nagel, Germany). Detected with SDS-PAGE analysis, the imidazole-eluted fractions containing rhCNTF were pooled, buffer exchanged and concentrated before final purification with size-exclusion chromatography (SEC). Pooled protein was loaded onto a Superdex 200 prep grade-packed C16/40 column and the elution, collection and handling of protein fractions carried out as described previously (Itkonen *et al.*, 2014). Purified protein was kept either on ice at 4 °C or snap-frozen with liquid N_2 for storage at -80 °C.

Table S1. Layout of the ThermoFluor buffer, salt, and pH screen.

	1	2	3	4	5	6	7	8	9	10	11	12
A	Na Acetate<br>pH 3.5	Na Acetate<br>50 mM NaCl<br>pH 3.5	Na Acetate<br>150 mM NaCl<br>pH 3.5	Na Acetate<br>500 mM NaCl<br>pH 3.5	HEPES<br>pH 7.5	HEPES<br>50 mM NaCl<br>pH 7.5	HEPES<br>150 mM NaCl<br>pH 7.5	HEPES<br>500 mM NaCl<br>pH 7.5	Na Acetate<br>pH 6.0	Na Acetate<br>50 mM NaCl<br>pH 6.0	Na Acetate<br>150 mM NaCl<br>pH 6.0	Na Acetate<br>500 mM NaCl<br>pH 6.0
B	Citric Acid<br>pH 4.0	Citric Acid<br>50 mM NaCl<br>pH 4.0	Citric Acid<br>150 mM NaCl<br>pH 4.0	Citric Acid<br>500 mM NaCl<br>pH 4.0	Imidazole<br>pH 8.0	Imidazole<br>50 mM NaCl<br>pH 8.0	Imidazole<br>150 mM NaCl<br>pH 8.0	Imidazole<br>500 mM NaCl<br>pH 8.0	Ammonium Acetate<br>pH 6.0	Ammonium Acetate<br>50 mM NaCl<br>pH 6.0	Ammonium Acetate<br>150 mM NaCl<br>pH 6.0	Ammonium Acetate<br>500 mM NaCl<br>pH 6.0
C	Ammonium Acetate<br>pH 4.5	Ammonium Acetate<br>50 mM NaCl<br>pH 4.5	Ammonium Acetate<br>150 mM NaCl<br>pH 4.5	Ammonium Acetate<br>500 mM NaCl<br>pH 4.5	Na-K Phosphate<br>pH 8.5	Na-K Phosphate<br>50 mM NaCl<br>pH 8.5	Na-K Phosphate<br>150 mM NaCl<br>pH 8.5	Na-K Phosphate<br>500 mM NaCl<br>pH 8.5	Na-K Phosphate<br>pH 6.5	Na-K Phosphate<br>50 mM NaCl<br>pH 6.5	Na-K Phosphate<br>150 mM NaCl<br>pH 6.5	Na-K Phosphate<br>500 mM NaCl<br>pH 6.5
D	Na Citrate<br>pH 5.0	Na Citrate<br>50 mM NaCl<br>pH 5.0	Na Citrate<br>150 mM NaCl<br>pH 5.0	Na Citrate<br>500 mM NaCl<br>pH 5.0	Bicine<br>pH 9.0	Bicine<br>50 mM NaCl<br>pH 9.0	Bicine<br>150 mM NaCl<br>pH 9.0	Bicine<br>500 mM NaCl<br>pH 9.0	MES<br>pH 7.0	MES<br>50 mM NaCl<br>pH 7.0	MES<br>150 mM NaCl<br>pH 7.0	MES<br>500 mM NaCl<br>pH 7.0
E	Na Citrate<br>pH 5.5	Na Citrate<br>50 mM NaCl<br>pH 5.5	Na Citrate<br>150 mM NaCl<br>pH 5.5	Na Citrate<br>500 mM NaCl<br>pH 5.5	CHES<br>pH 9.5	CHES<br>50 mM NaCl<br>pH 9.5	CHES<br>150 mM NaCl<br>pH 9.5	CHES<br>500 mM NaCl<br>pH 9.5	Na Phosphate<br>50 mM NaCl<br>pH 7.0	Na Phosphate<br>300 mM NaCl<br>pH 7.0	Imidazole<br>pH 6.5	Imidazole<br>150 mM NaCl<br>pH 6.5
F	MES<br>pH 6.0	MES<br>50 mM NaCl<br>pH 6.0	MES<br>150 mM NaCl<br>pH 6.0	MES<br>500 mM NaCl<br>pH 6.0	CAPS<br>pH 10.5	CAPS<br>50 mM NaCl<br>pH 10.5	CAPS<br>150 mM NaCl<br>pH 10.5	CAPS<br>500 mM NaCl<br>pH 10.5	Triethanolamine	Triethanolamine<br>50 mM NaCl	Na-K Phosphate<br>pH 7.0	Na-K Phosphate<br>150 mM NaCl<br>pH 7.0
G	MES<br>pH 6.5	MES<br>50 mM NaCl<br>pH 6.5	MES<br>150 mM NaCl<br>pH 6.5	MES<br>500 mM NaCl<br>pH 6.5	Na Acetate<br>pH 4.5	Na Acetate<br>50 mM NaCl<br>pH 4.5	Na Acetate<br>150 mM NaCl<br>pH 4.5	Na Acetate<br>500 mM NaCl<br>pH 4.5	Na Acetate<br>pH 5.5	Na Acetate<br>150 mM NaCl<br>pH 5.5	Ammonium Citrate<br>pH 7.5	Ammonium Citrate<br>150 mM NaCl<br>pH 7.5
H	HEPES<br>pH 7.0	HEPES<br>50 mM NaCl<br>pH 7.0	HEPES<br>150 mM NaCl<br>pH 7.0	HEPES<br>500 mM NaCl<br>pH 7.0	Na Acetate<br>pH 5.5	Na Acetate<br>50 mM NaCl<br>pH 5.5	Na Acetate<br>150 mM NaCl<br>pH 5.5	Na Acetate<br>500 mM NaCl<br>pH 5.5	Na Citrate<br>pH 5.6	Na Citrate<br>150 mM NaCl<br>pH 5.6	Imidazole/ Maleic acid<br>pH 8.5	Imidazole/ Maleic acid<br>150 mM NaCl<br>pH 8.5

All buffers used at 100 mM concentration.

Table S2. Tissue processing procedure.

Solvent	Time
Tap water	10 minutes
80% ethanol	30 minutes
94% ethanol	30 minutes
94% ethanol	30 minutes
99% ethanol	45 minutes
99% ethanol	30 minutes
99% ethanol	30 minutes
Xylene	20 minutes
Xylene	20 minutes
Liquid paraffin	1 hour
Liquid paraffin	1 to 24 hours

Table S3. H&E staining protocol.

Reagent	Time
Xylene	2 x 5 minutes
100 % ethanol	2 x 2 minutes
94 % ethanol	2 x 2 minutes
Rinsing with distilled water	20 seconds
Delafield hematoxylin	9 minutes
Rinsing with tap water	5 minutes
1 % HCl in 70% ethanol	4-5 seconds
Rinsing with tap water	10 minutes
1 % eosin	30 seconds
94 % ethanol	2 x 2 minutes
100 % ethanol	2 x 2 minutes
Xylene	2 x 5 minutes

Xylene (BDH Prolabo, VWR Chemicals, France), and 100% and 94% ethanol (Altia Oyj, Finland) were purchased from manufacturers. Delafield hematoxylin, 1% HCl in 70% ethanol and 1% eosin were prepared in the University of Eastern Finland. Prior staining, the 1% eosin was filtered and 1 % of glacial acetic acid (BDH Prolabo, VWR Chemicals, France) was added.

Immunofluorescence staining of rat retinal explants

Immunofluorescence staining was carried out to assess the localization of labeled rhCNTF. Slides with fixed sections of retinal tissue were washed 3 times with PBS followed by incubation with blocking buffer (10% goat serum, 1% BSA, 0.5% Triton-X) for 1 h at room temperature. Next, samples were incubated with primary polyclonal antibody against Iba-1 (1:200, FUJIFILM Wako Chemicals U.S.A. Corp), a specific microglial marker, diluted in IHC antibody buffer (3% goat serum, 1% BSA, 0.5% Triton-X) overnight at 4 °C. Next day, the slides were washed 3 times for 5 min with PBS and incubated with Alexa Fluor™ 568-conjugated secondary antibody (1:350, Thermo Fisher Scientific) diluted in IHC antibody buffer for 1 h at room temperature. Further, to visualize cell nuclei, counterstaining was carried out by incubating the slides in 4′,6-diamidino-2-pphenylindole (DAPI) diluted in PBS (1:5000) for 5 min. Finally, samples were washed 3 times for 5 min with PBS, dried out, embedded with Fluoromount-G™ antifade reagent (Thermo Fisher Scientific), and closed with cover glasses.

Table S4. Heat map of rhCNTF T_h measured in ThermoFluor screen.

Cells with underlined results indicate buffers chosen for further studies.

	1	2	3	4	5	6	7	8	9	10	11	12
A	37.3	27.4	26.3	26.3	54.2	54.2	56.2	57.2	53.2	54.2	55.2	57.2
B	45.2	45.2	45.2	41.2	52.2	53.2	54.2	59.3	52.2	54.2	54.2	55.2
C	43.2	41.2	41.2	43.2	53.2	56.2	55.2	57.2	53.2	54.2	56.2	56.2
D	53.2	54.2	52.2	51.2	51.2	52.2	54.2	56.2	52.2	53.2	55.2	59.2
E	58.2	57.2	56.2	53.2	52.2	51.2	52.2	54.2	56.2	*	50.2	52.2
F	50.2	50.2	51.2	52.2	38.3	43.2	43.3	45.2	54.2	56.2	56.2	57.2
G	51.2	52.2	53.2	55.2	42.2	42.2	43.2	43.2	*	50.2	56.2	58.2
H	52.2	53.2	54.2	56.2	46.2	47.2	47.2	46.2	58.2	56.2	53.2	54.2

Blue corresponds to the lowest T_h and red to the highest T_h.

*No discernible peak on derivative plot, no T_h determined.

Table S5. R_h estimation of rhCNTF stored on ice at +4°C.

	Days post-purification		2	14	28	48	52
**buffer M**	Size						
	Monomer/dimer	R_h ± SD (nm)	3.44 ± 0.21	3.45 ± 0.11	3.52 ± 0.53	3.83 ± 0.25	2.86 ± 0.18
		Peak PdI	0.02	0.07	0.04	0.11	0.02
	HMW oligomers	R_h ± SD (nm)	N/A	66 ± 24	129 ± 87	31 ± 0	48 ± 33
	Relative % of HMW						
	By intensity distribution		N/A	22.6 ± 2.5	11.8 ± 9.5	19.9 ± 0.0	45.1 ± 28.2
	By volume distribution		0	< 0.7	< 0.6	0	< 0.2

	Days post-purification		2	14	28	48	52
**buffer C**	Size						
	Monomer/dimer	R_h ± SD (nm)	2.99 ± 0.2	2.89 ± 0.04	2.85 ± 0.20	3.16 ± 0.25	2.91 ± 0.08
		Peak PdI	0.01	0.03	0.02	0.05	0.03
	HMW oligomers	R_h ± SD (nm)	N/A	68 ± 8	45 ± 14	138 ± 49	96 ± 43
	Relative % of HMW						
	By intensity distribution		N/A	N/A	12.4 ± 0.0	14.4 ± 0.6	21.6 ± 9.7
	By volume distribution		0	0	0	0	0

HMW oligomers R_h 30-200 nm.

Table S6. R_h estimation of rhCNTF stored at -80°C.

buffer M

A) Thawed sample; unmixed and uncentrifuged

Days post-thaw			12	25	26	45	46
Size							
	Monomer/dimer	R_h ± SD (nm)	2.69 ± 0.09	3.82 ± 0.22	3.43 ± 0.33	2.68 ± 0.53	4.04 ± 0.02
		Peak PdI	0.05	0.06	0.04	0.11	0.06
	HMW oligomers	R_h ± SD (nm)	91.0 ± 16	57.0 ± 42	95 ± 21	79 ± 48	92 ± 49
Relative % of HMW							
	By intensity distribution		14.5 ± 4.2	12.3 ± 3.4	15.4 ± 0.4	57.0 ± 27.8	11.5 ± 1.0
	By volume distribution		0	< 0.3	< 0.8	< 0.1	0

B) Thawed sample; centrifugally cleared supernatant

Days post-thaw			12	25	26	45	46
Size							
	Monomer/dimer	R_h ± SD (nm)	2.55 ± 0.25	3.56 ± 0.30	3.63 ± 0.20	2.62 ± 0.39	3.87 ± 0.25
		Peak PdI	0.12	0.09	0.06	0.09	0.09
	HMW oligomers	R_h ± SD (nm)	N/A	N/A	113 ± 0	89 ± 17	114 ± 0
Relative % of HMW							
	By intensity distribution		N/A	17.2 ± 0.0	5.8 ± 0.0	34.7 ± 9.4	6.2 ± 0.0
	By volume distribution		0	0	0	0	0

buffer C

A) Thawed sample; unmixed and uncentrifuged

Days post-thaw			10	12	24	25	26	45	46
Size									
	Monomer/dimer	R_h ± SD (nm)	3.02 ± 0.37	3.17 ± 0.17	2.66 ± 0.25	3.33 ± 0.44	2.92± 0.05	3.18 ± 0.16	3.19 ± 0.16
		Peak PdI	0.05	0.01	0.09	0.07	0.03	0.04	0.03
	HMW oligomers	R_h ± SD (nm)	113 ± 78	162 ± 1	143 ± 45	N/A	95 ± 21	118 ± 64	109 ± 34
Relative % of HMW									
	By intensity distribution		12.7 ± 4.4	19.6 ± 12.2	16.5 ± 4.3	N/A	15.4 ± 0.4	20.2 ± 3.2	18.3 ± 5.3
	By volume distribution		0	0	0	0	0	0	0

B) Thawed sample; centrifuged cleared supernatant

Days post-thaw			10	12	24	25	26	45	46
Size									
	Monomer/dimer	R_h ± SD (nm)	2.89 ± 0.4	3.02 ± 0.04	2.84 ± 0.14	2.92 ± 0.11	3.27 ± 0.05	3.08 ± 0.31	3.33 ± 0.33
		Peak PdI	0.08	0.03	0.04	0.09	0.02	0.04	0.07
	HMW oligomers	R_h ± SD (nm)	N/A	99 ± 70	80 ± 58	N/A	81 ± 4	109 ± 54	N/A
Relative % of HMW									

By intensity distribution	N/A	16.0 ± 6.6	15.2 ± 3.7	N/A	32.3 ± 3.5	18.9 ± 9.6	N/A
By volume distribution	0	< 0.1	0	0	0	0	0

HMW oligomers R_h 30-200 nm.

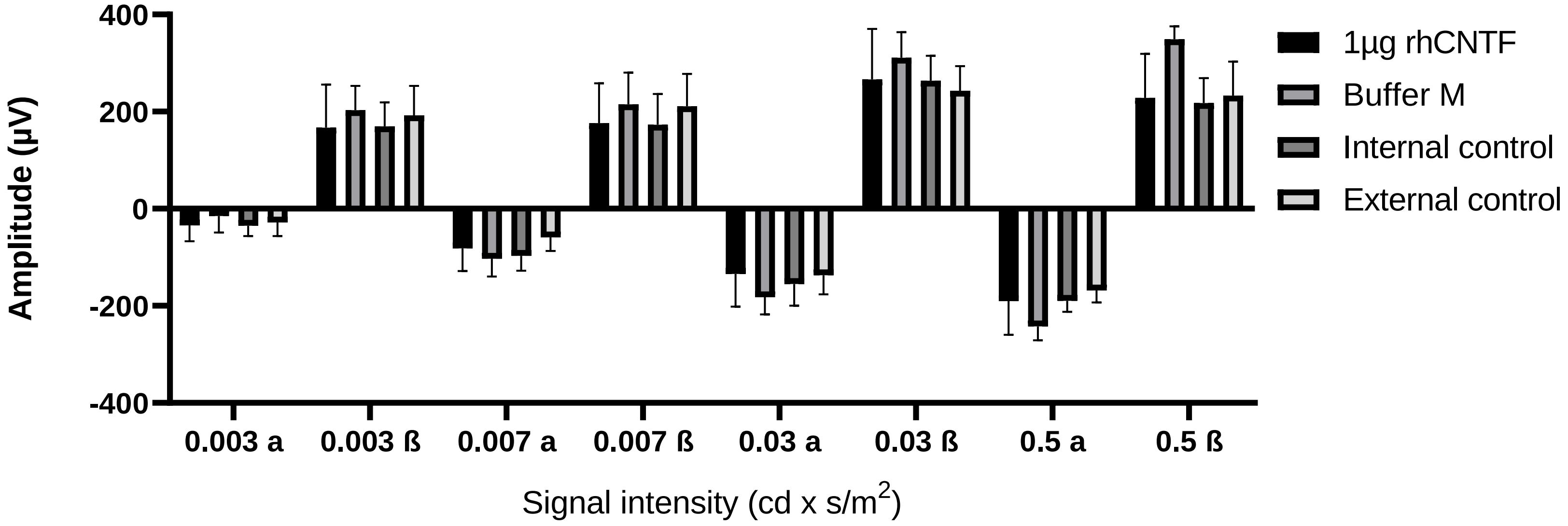

Figure S1. Mean and SD of recorded scotopic α and β wave amplitudes in the 2nd study set 1 week after intravitreal injection (n=6).

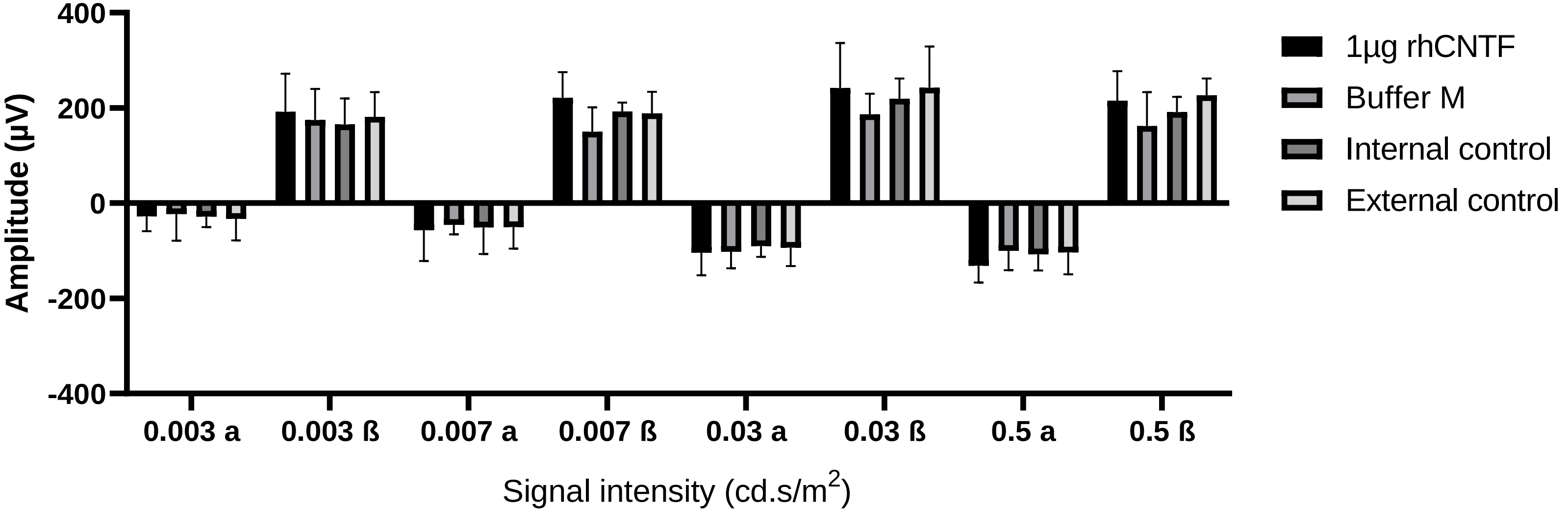

Figure S2. Mean and SD of recorded scotopic α and β wave amplitudes in the 2nd study set 2 weeks after intravitreal injection (n=6).

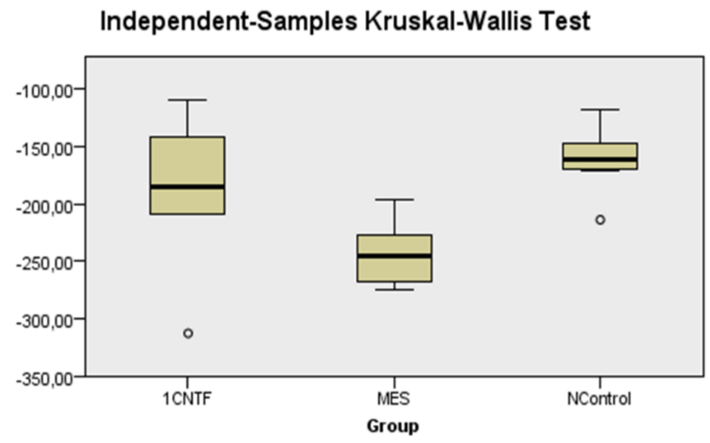

Figure S3. Left eye α wave distribution 1 week post-injection, scotopic 0.05 cd × s/m² ERG. The α wave amplitudes were significantly lower in the MES treated group than in the NControl group (Kruskal-Wallis test, p = 0.016). The NControl group α wave values did not statistically significantly differ from the 1 μg CNTF treated group (Kruskal-Wallis test, p = 0.411).

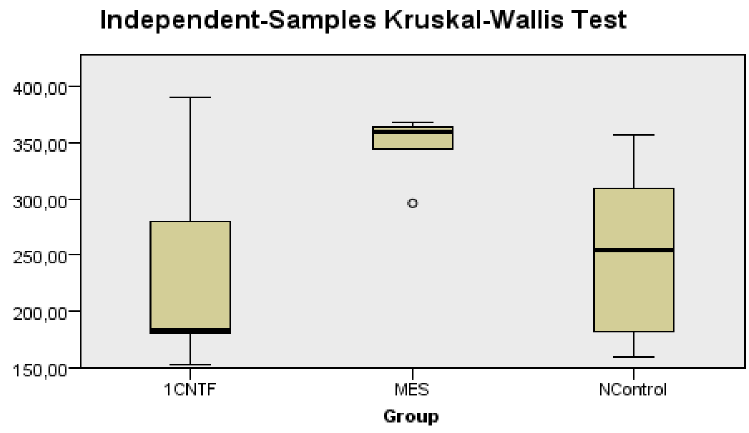

Figure S4. Left eye β wave distribution 1 week post-injection, scotopic 0.5 cd × s/m² ERG. left eye β wave amplitudes were significantly higher in the MES treated group than in 1 μg CNTF treated group (Kruskal-Wallis test, p = 0.018) and NControl group (p = 0.025). However, the 1 μg CNTF treated group and the NControl group did not show any statistically significant difference between their mean values (p = 0.831).

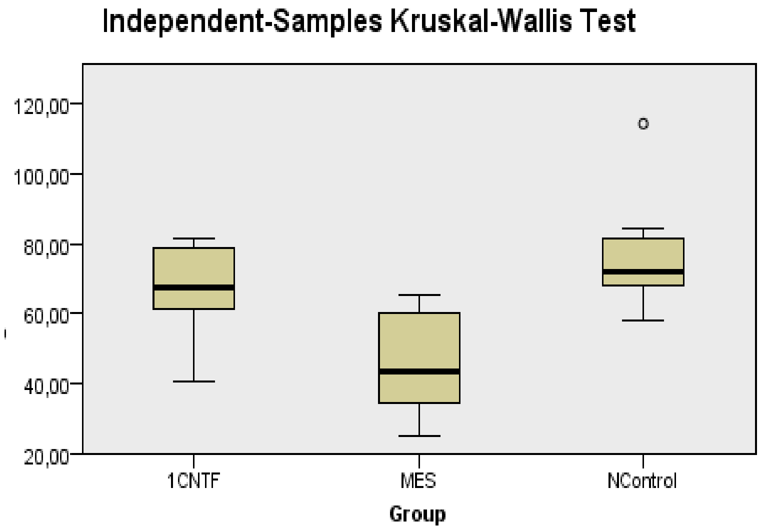

Figure S5. Left eye β wave distribution 2 weeks post-injection, photopic 1 cd × s/m^2 ERG. Left eye β wave amplitudes were significantly higher in the NControl group than in MES treated group (Kruskal-Wallis test, p =0.007). The difference between 1 μg CNTF group and MES group was not statistically significant (p = 0.058).

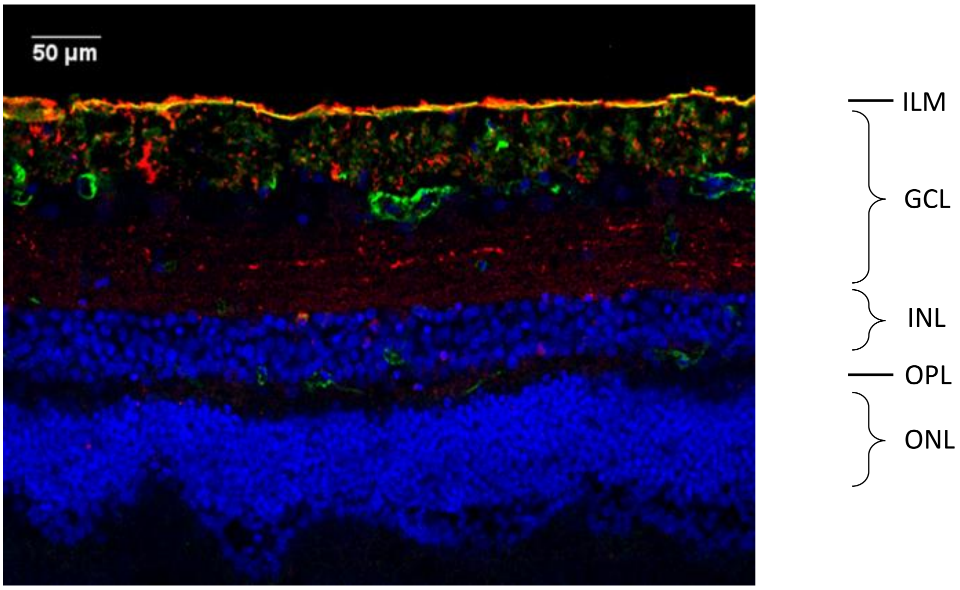

Figure S6. rhCNTF penetration in bovine retinal explant. NT-647 labeled rhCNTF (red) readily penetrates into the retina after apical administration, with fluorescence observed in the neural retina in layers ranging from GCL to OPL, and even the ONL. ILM, inner limiting membrane; GCL, ganglion cell layer; ILM, inner limiting membrane; IPL, Inner plexiform layer; INL, inner nuclear layer; OPL, outer plexiform layer; ONL, outer nuclear layer; OPL, outer plexiform layer.

Calculations of intravitreal half-life of CNTF based on R_h

Based on the comprehensive collection of the intravitreal pharmacokinetic parameters of volume of distribution ($V_{ss,ivt}$) and clearance (CL_{ivt}) of intravitreal biologicals in rabbit and human eye (del Amo *et al.*, 2015; del Amo & Urtti 2016) the following calculations were done:

1. A linear correlation between R_h (Shatz *et al.*, 2016) and CL_{ivt} that allowed the calculation of rabbit CL_{ivt} CNTF.

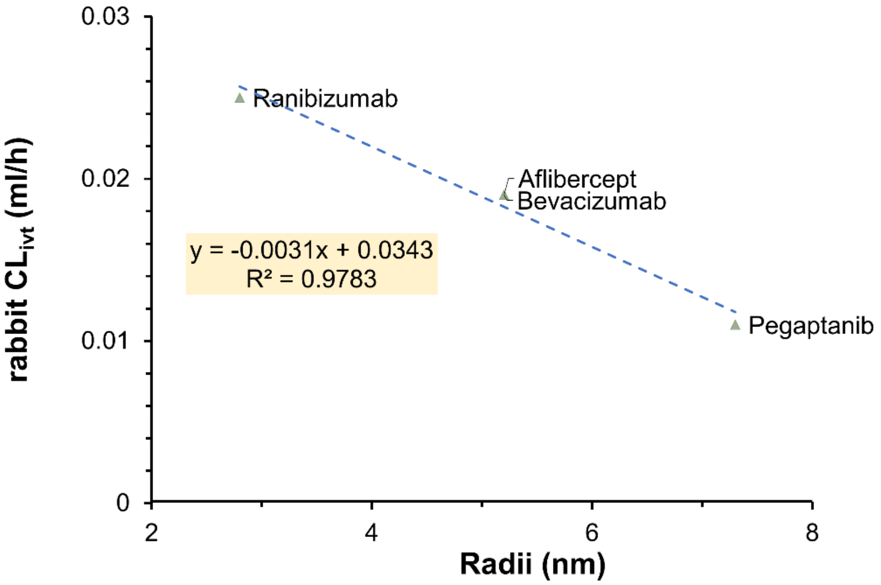

Based on its R_h of 2.95 nm and 3.32 nm, rabbit CL_{ivt} CNTF are expected to be 0.025 ml/h and 0.024 ml/h respectively.

2. Human $V_{ss,ivt}$ and CL_{ivt} approximates four and two times the rabbit $V_{ss,ivt}$ and CL_{ivt} respectively. Therefore, human CNTF CL_{ivt} are predicted to be 0.050 ml/h and 0.047 ml/h for each R_H which correspond to half-lives of 4.68 and 4.97 days (based on the average rabbit $V_{ss,ivt}$ of 2.04 ml for macromolecules, the expected human $V_{ss,ivt}$ is 8.16 ml).

References

del Amo, E.M.; Vellonen, K.-S.; Kidron, H.; Urtti, A. Intravitreal clearance and volume of distribution of compounds in rabbits: In silico prediction and pharmacokinetic simulations for drug development. *Eur. J. Pharm. Biopharm.* **2015**, *95*, 215-26

del Amo, E.M.; Urtti, A. Computational Prediction of Intravitreal Pharmacokinetics of Macromolecules: Tool for Ocular Drug Development. *Invest. Ophthalmol. Vis. Sci.* **2016**, *ARVO abstract 57*(12), 291.

Itkonen, J.M.; Urtti, A.; Bird, L.E.; Sarkhel, S. Codon optimization and factorial screening for enhanced soluble expression of human ciliary neurotrophic factor in *Escherichia coli*. *BMC Biotechnol.* **2014**, *14*,92. doi: 10.1186/s12896-014-0092-x

Shatz, W.; Hass, P.E.; Mathieu, M.; Kim, H.S.; Leach, K.; Zhou, M.; *et al.* Contribution of antibody hydrodynamic size to vitreal clearance revealed through rabbit studies using a species-matched fab. *Mol. Pharm.* **2016**, *13*(9), 2996-3003. doi: 10.1021/acs.molpharmaceut.6b00345
